# Novel CRISPR-based sequence specific enrichment methods for target loci and single base mutations

**DOI:** 10.1371/journal.pone.0243781

**Published:** 2020-12-23

**Authors:** Jennifer L. Steele, Richard C. Stevens, Oscar A. Cabrera, Gary J. Bassill, Sabrina M. Cramer, Felipe Guzman, Anthony P. Shuber

**Affiliations:** Genetics Research LLC, Waltham, Massachusetts, United States of America; University of Helsinki, FINLAND

## Abstract

The programmable sequence specificity of CRISPR has found uses in gene editing and diagnostics. This manuscript describes an additional application of CRISPR through a family of novel DNA enrichment technologies. CAMP (CRISPR Associated Multiplexed PCR) and cCAMP (chimeric CRISPR Associated Multiplexed PCR) utilize the sequence specificity of the Cas9/sgRNA complex to target loci for the ligation of a universal adapter that is used for subsequent amplification. cTRACE (chimeric Targeting Rare Alleles with CRISPR-based Enrichment) also applies this method to use Cas9/sgRNA to target loci for the addition of universal adapters, however it has an additional selection for specific mutations through the use of an allele-specific primer. These three methods can produce multiplex PCR that significantly reduces the optimization required for every target. The methods are also not specific to any downstream analytical platform. We additionally will present a mutation specific enrichment technology that is non-amplification based and leaves the DNA in its native state: TRACE (Targeting Rare Alleles with CRISPR-based Enrichment). TRACE utilizes the Cas9/sgRNA complex to sterically protect the ends of targeted sequences from exonuclease activity which digests both the normal variant as well as any off-target sequences.

## Introduction

The application of the CRISPR (Clustered Regularly Interspaced Short Palindromic Repeats) system to gene editing promises to revolutionize both the life science and medical fields [1–3]. While much focus has been placed on the *in vivo* applications of the CRISPR system, due to the unique programmable nature of the enzymes, there are also several *in vitro* applications for this system; for example, several recent publications report its use in diagnostics [4–7]. In a previous publication, we described an additional *in vitro* application of the CRISPR system to enrich long (10–36 kb) targets of native DNA using Negative Enrichment [8]. Negative Enrichment is based on the protection of specific loci from exonuclease digestion using *Streptococcus pyogenes* Cas9 nuclease (Cas9) complexed with a single guide RNA (Cas9/sgRNA). Here, we report an additional application of CRISPR that provides amplification-based enrichment to target short sequences and allele-specific enrichment to target rare mutations from both long cellular DNA and circulating cell-free DNA (cfDNA).

Targeted enrichment methods are needed to both increase the sensitivity to detect rare mutations and to better produce rapid cost-effective clinical results on various analytical platforms [9]. Recent advances in both sequencing and non-sequencing DNA analysis technologies will improve and expand clinical diagnostic assays [10–12]. However, these technological strides forward will require equivalent advancements in sample preparation methods in order to effectively move from the laboratory to the clinic [13]. This is particularly evident in the analysis of cfDNA for the early detection of cancer and cancer recurrence monitoring. The use of cfDNA for clinical diagnostics presents a challenge as it is typically in quantities less than 100 ng/mL of blood, the mutational frequency associated with circulating tumor DNA (ctDNA) is low, and the fragment size of the DNA is small [14–22].

Current common methods for targeted enrichment [23–29] include both hybrid capture and sequence specific PCR. Hybrid capture is a probe-based method that utilizes overlapping single-strand oligonucleotides for positive enrichment to capture targeted DNA fragments. In general, it has inconsistent efficiencies across many targets that require amplifications to improve the yield for analysis [30,31]. Sequence specific PCR uses amplification to increase the signal of targeted DNA, however the ability to analyze multiple targets simultaneously while retaining the signal to background ratios that can be achieved with single PCR reactions is limited and requires extensive optimization [32–34].

Commonly used methods for mutation specific enrichment are also primarily amplification-based; for example, in allele-specific PCR (ASP) the specificity of the primer is used to enrich mutations while normal variants are not amplified [35–40]. PCR-based mutation specific enrichment is difficult to multiplex to many relevant biomarkers. This amplification involves careful design of primers and conditions to give discretion between mutant and normal variants, and the conditions must be optimized for each site of interest [40].

Other approaches to the analysis of clinically relevant mutations involve whole genome sequencing, whole exome sequencing, or targeted gene panels with no specificity for the mutant allele [41]. Whole genome sequencing and whole exome sequencing can generate comprehensive assays of genetic variants, however, there is significant cost and time associated with the analysis of the entire genome [9,42]. Targeted gene panels can be inconsistent in detecting rare mutations; for example, in a recent study four Next Generation Sequencing (NGS) gene panel assays were compared and found to be variable for the analysis of ctDNA particularly in samples with less than one percent mutational frequency [43].

In addition to these standard targeted and mutation enrichment technologies, several published articles have recently described other CRISPR-based enrichment techniques for sequencing libraries. Depletion of Abundant Sequences by Hybridization (DASH) removes off-target sequences by treating an NGS library with Cas9 complexes to target background sequences; this allows only the uncut species to be analyzed by NGS [44,45]. Another CRISPR-based method, Finding Low Abundance Sequences by Hybridization (FLASH), involves a dephosphorylating pre-treatment of the input DNA and use of Cas9 to generate targeted phosphorylated ends necessary for library preparation [46]. Similar techniques, combining dephosphorylation with Cas9-based enrichment have also been used with the Oxford Nanopore Sequencing platform for long DNA applications [47–49], along with several other CRISPR-based enrichment techniques for long DNA sequencing platforms [50,51]. There have also been reports of using Cas9/sgRNA for positive enrichment [52,53].

Herein, we report a series of novel CRISPR-based DNA enrichment technologies for the targeted enrichment of short loci and specific mutations (S1 Fig). For targeted enrichment, these amplification-based DNA enrichment techniques utilize Cas9/sgRNA complexes to flank specific target sequences based on chosen guide RNAs and to ligate common adapters for subsequent amplification. In CRISPR Associated Multiplex PCR (CAMP) the amplification is completed using a single universal priming sequence (UPS) complementary to the UPS adapter. In chimeric-CRISPR Associated Multiplex PCR (cCAMP) amplification is completed using a primer [54] that contains both a region complementary to the UPS adapter as well as several bases of sequence specific target providing improved specificity.

We also report two mutation specific enrichment methodologies: Targeting Rare Alleles with CRISPR-based Enrichment (TRACE) and chimeric Targeting Rare Alleles with CRISPR-based Enrichment (cTRACE). TRACE is based on Negative Enrichment [8] and does not include any amplification steps. Negative Enrichment uses the long residence time of Cas9/sgRNA [55,56] to provide the steric inhibition from exonuclease which digests the DNA outside of the target loci. In TRACE the sgRNA is designed to match a single base mutation, which is protected while the normal variant and the background DNA are digested by exonuclease. cTRACE uses a similar amplification-based methodology to cCAMP through the ligation of UPS adapters, however, it then uses a chimeric primer with a mutation specific 3’-end to enrich the mutated allele over the normal variant.

These methods do not require the significant optimization of reaction conditions for each target site associated with the standard approach of multiplexing PCR or ASP reactions. Thus, CAMP, cCAMP, and cTRACE demonstrate the capability to produce a multiplexed PCR or multiplex ASP that amplifies any number of biomarker DNA targets that are clinically relevant. These methods are also not specific to any platform and can be used to enrich DNA for various downstream analytical output.

## Materials and methods

### Demonstration of multiplexed enrichment with CAMP on long human genomic DNA

The sgRNAs used are listed in S1 Table. Pairs of sgRNAs for each of the human genomic targets were combined and bound to Cas9 Nuclease (New England Biolabs (NEB), cat. #M0386M) for 30 minutes at 25°C in 1X Cas9 buffer (20 mM HEPES, 100 mM NaCl, 5 mM MgCl2, 0.1 mM EDTA, pH 6.5). Samples were then diluted with a mixture of 1X NEBuffer 1 (NEB, cat. #B7001S) and 1 mM Adenosine 5'-Triphosphate (NEB, cat. #P0756S) and mixed with 20 ng human genomic DNA (Promega, cat. #G3041), incubated for 60 minutes at 37°C, and purified. To purify, samples were phenol-chloroform extracted, ethanol precipitated using standard techniques, and resuspended in 10 mM Tris, pH 7.5.

Next, samples were treated with the NEBNext dA-Tailing Module (NEB, cat. #E6053L) with additional dNTPs (dCTP, dGTP, dTTP) (NEB, cat. #N0446S) added, and UPS adapters were ligated (S2 Table) using the NEBNext Ultra II Ligation Module (NEB, cat. #E7595L). DNA was purified using AMPure XP beads (Beckman Coulter, cat. #A63881) and resuspended.

CAMP amplification was performed using a universal primer (S2 Table) and LongAmp Hot Start Taq Polymerase (NEB, cat. #M0534L) with an annealing temperature of 65˚C. After amplification, DNA was purified using AMPure XP beads (Beckman Coulter, cat. #A63881) and resuspended in 10 mM Tris, pH 7.5. Agarose gel analysis was performed on an aliquot of each reaction with a 2.0% LE agarose gel (Lonza, cat. #50004) in TAE (Boston BioProducts, cat. #BM-250) for 1.5 hours at 96 volts on a Gibco-BRL Gel Electrophoresis Apparatus (cat. #21087–010) with GelRed (Biotium, cat. #41003) and imaged with a Nikon D3000 digital camera using a Stratagene 2020E Transilluminator.

Further analysis of the products was performed by qPCR and enrichment ratios were quantified using FAM-based probes (IDT, S3 Table) following the manufacturer’s instructions. The qPCR analyses were performed using a Rotor-Gene Q (Qiagen, cat. #9001550) and the QuantiNova Probe PCR Kit (Qiagen, cat. #208252) following the manufacturer’s instructions.

### Demonstration of multiplexed enrichment with cCAMP on long human genomic DNA and a cfDNA model

The sgRNAs used are listed in S1 Table. Pairs of sgRNAs for each of the human genomic targets were combined and bound to Cas9 Nuclease (NEB, cat. #M0386M) for 30 minutes at 25°C in 1X Cas9 buffer (20 mM HEPES, 100 mM NaCl, 5 mM MgCl2, 0.1 mM EDTA, pH 6.5). Samples were then diluted with a mixture of 1X NEBuffer 1 (NEB, cat. #B7001S) and 1 mM Adenosine 5'-Triphosphate (NEB, cat. #P0756S) and mixed with either 20 ng human genomic DNA (Promega, cat. #G3041) or 100 ng of cfDNA model, which was prepared by shearing genomic DNA with a Covaris M-220 ultra-sonicator to an average size of 166 bp. The solution was then incubated for 60 minutes at 37°C and purified by phenol-chloroform extraction and ethanol precipitation and resuspended in 10 mM Tris, pH 7.5.

Next, the samples were treated with Klenow (exo-) (NEB, cat. #M0212L) in KAPA HyperPlus End Repair & A-Tailing Buffer (Kapa Biosystems, cat. #KK8515). UPS adapters were ligated (S2 Table) using the KAPA HyperPlus Ligation Buffer and Ligase Enzyme. DNA was purified using AMPure XP beads (Beckman Coulter, cat. #A63881) and resuspended. Alternatively, the repair and ligation steps can be completed with the NEBNext dA-Tailing Module (NEB, cat. #E6053L) with the addition of dCTP, dGTP, and dTTP (NEB, cat. #N0446S) and NEBNext Ultra II Ligation Module (NEB, cat. #E7595L).

cCAMP amplification was performed using chimeric primers with a 6-base target specificity for a long DNA input and 10-base target specificity for the cfDNA model (S2 Table). LongAmp Hot Start Taq Polymerase (NEB, cat. #M0534L) was used with an annealing temperature of 65˚C (long DNA) or 72°C (cfDNA). After amplification, DNA was purified using AMPure XP beads (Beckman Coulter, cat. #A63881) and resuspended in 10 mM Tris, pH 7.5. Agarose gel analysis was performed as described for CAMP above.

Two sequence specific PCR amplifications were performed in the target regions for comparison to cCAMP. Primers used are listed in S2 Table, and cCAMP PCR conditions were used.

### Demonstration of mutation specific enrichment with TRACE (Post-PCR)

The sgRNAs used are listed in S1 Table. A single sgRNA was selected with the desired matched or mismatched sequence and bound to Cas9 Nuclease (NEB, cat. #M0386M) for 30 minutes at 25°C. The Cas9/sgRNA complexes were then mixed with 150 ng of PCR product (S1 Table) and incubated for 60 minutes at 37°C. Next, lambda exonuclease (NEB, cat. #M0262L) and exonuclease VII (NEB, cat. #M0379L) were added with 1X lambda exonuclease buffer and incubated for a total of 120 minutes at 37°C.

Agarose gel analysis was performed on an aliquot of each reaction on a 2% LE agarose gel (Lonza, cat. #50004) in TAE (Boston BioProducts, cat. #BM-250) for two hours at 96 volts on a Gibco-BRL Gel Electrophoresis Apparatus (cat. #21087–010) with GelRed (Biotium, cat. #41003) and imaged with a Nikon D3000 digital camera using a Stratagene 2020E Transilluminator.

### Demonstration of non-amplification-based mutation specific enrichment with TRACE from long human genomic DNA and a cfDNA model

The sgRNAs used are listed in S1 Table. sgRNAs were bound to Cas9 Nuclease (NEB, cat. #M0386M) for 30 minutes at 25°C. The Cas9/sgRNA complexes were then mixed with target DNA and incubated for 60 minutes at 37°C. Next, exonuclease III (NEB, cat. #M0206L) and exonuclease VII (NEB, cat. #M0379L) were added with NEBuffer 1 (NEB, cat. #B7001S) and incubated for a total of 240 minutes at 37°C. Genomic DNA experiments used 200 ng DNA (Horizon Discovery, cat. #HD272; Promega cat. #G3041) and cfDNA enrichment experiments used 200 ng of Horizon Discovery Multiplex I DNA (cat. #HD780). Samples were phenol-chloroform extracted and ethanol precipitated using standard techniques. Samples were resuspended in 10 mM Tris, pH 7.5 before further analysis.

### Demonstration of enrichment with cTRACE on human genomic DNA

The sgRNAs used are listed in S1 Table. Pairs of sgRNAs for each of the human genomic targets were combined and bound to Cas9 Nuclease (NEB, cat. #M0386M) for 30 minutes at 25°C in 1X Cas9 buffer (20 mM HEPES, 100 mM NaCl, 5 mM MgCl2, 0.1 mM EDTA, pH 6.5). Samples were then diluted with a mixture of 1X NEBuffer 1 (NEB, cat. #B7001S) and 1 mM Adenosine 5'-Triphosphate (NEB, cat. #P0756S) and mixed with 20 ng human genomic DNA (Promega, cat. #G3041), incubated for 60 minutes at 37°C, and purified as above.

Next, samples were treated with Klenow (exo-) (NEB, cat. #M0212L) in KAPA HyperPlus End Repair & A-Tailing Buffer (Kapa Biosystems, cat. #KK8515). UPS adapters were ligated (S2 Table) using the KAPA HyperPlus Ligation Buffer and Ligase Enzyme. DNA was purified using AMPure XP beads (Beckman Coulter, cat. #A63881) and resuspended.

cTRACE amplification was performed using chimeric primers (S2 Table) and LongAmp Hot Start Taq Polymerase (NEB, cat. #M0534L), with an annealing temperature of 65˚C. After amplification, DNA was purified using AMPure XP beads (Beckman Coulter, cat. #A63881) and resuspended in 10 mM Tris, pH 7.5. Agarose gel analysis was performed as described for CAMP above.

### Generation of DNA libraries and Illumina sequencing

Illumina sequencing libraries were prepared using the Kapa HyperPlus Kit (Kapa Biosystems, cat. #KK8514) following the manufacturer’s instructions. Libraries were sequenced on a NextSeq 550 system (Illumina, Inc) with all samples run as paired-end 150 bp reads. Reads were mapped to the respective target genomes (human build GRCh38/hg38) using BWA (Burrows-Wheeler Aligner, version 0.7.12-r1039) [57]. For human genomic samples, reads were mapped to the GRCh38 human genome reference assembly, using BWA-MEM. The alignments were sorted and indexed using SAMtools (version 1.3.1) [58]. Coverage graphs were generated for each of the subsets using BEDTools (version 2.27.1) [59], which were used to create coverage plots using the UCSC Genome Browser [60].

## Results and discussion

### Demonstration of multiplexed enrichment with CAMP on long human genomic DNA

**Fig 1:**
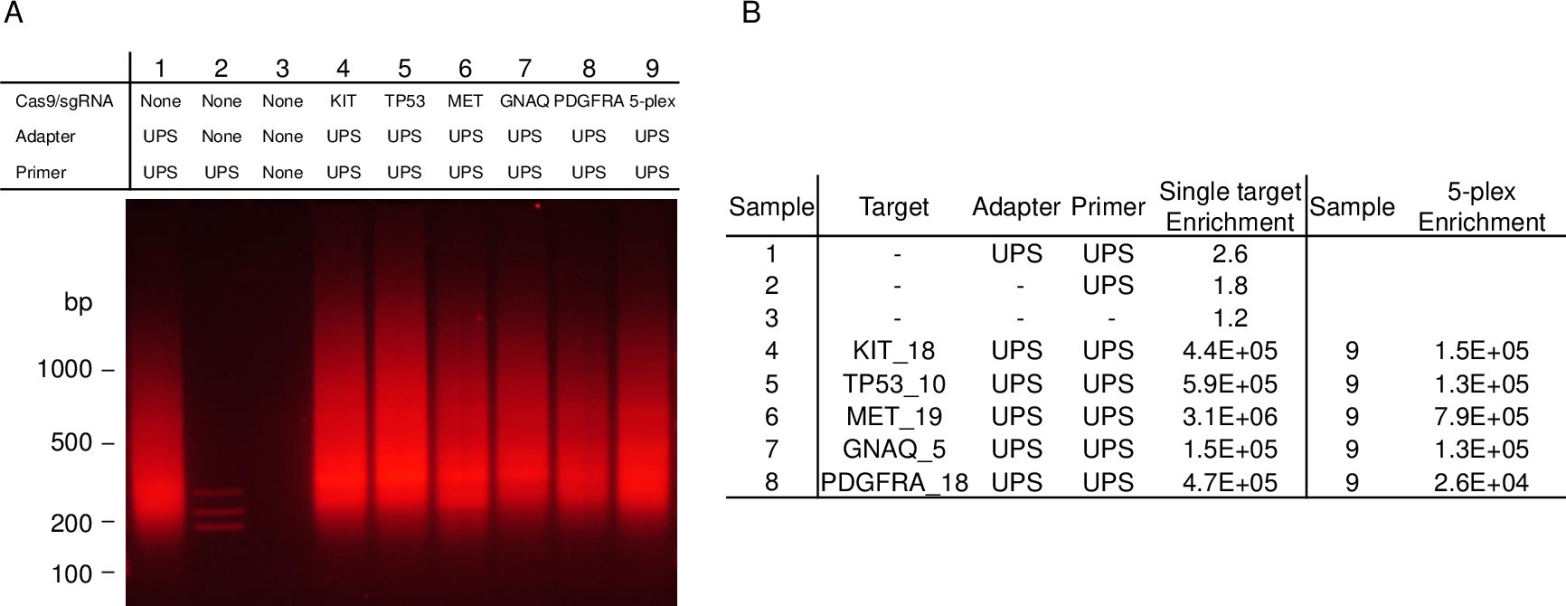
Gel electrophoresis and qPCR results of enrichment from CAMP on five single targets and a 5-plex. (A) Gel electrophoresis results from CAMP targeting loci in *KIT* exon 18 (Lane 4), *TP53* exon 10 (Lane 5), *MET* exon 19 (Lane 6), *GNAQ* exon 5 (Lane 7), and *PDGFRA* exon 18 (Lane 8). Results of the 5-plex of these targets is shown in Lane 9. Controls are shown in Lanes 1–3: Lane 1 shows reaction without Cas9/sgRNA but with UPS adapter and UPS primer, Lane 2 shows reaction without Cas9/sgRNA and without adapter but with UPS primer, and Lane 3 shows reaction without Cas9/sgRNA, UPS primer, and UPS adapter. (B) qPCR results from CAMP on the five individual targets and 5-plex. Enrichment was calculated by dividing the qPCR value for each target by an off-target qPCR value and then normalizing to a DNA standard sample. Enrichment for Samples 1–3 are averages for the five individual targets.

To investigate the use of CRISPR to target short DNA loci for enrichment, we first developed CAMP. CAMP utilizes the sequence specificity of Cas9/sgRNA (S1 Table) to target loci for the ligation of UPS adapters and amplification using a universal primer that has complementarity to the UPS adapter (S2 Table). Fig 1A shows the results of CAMP targeting five loci in *KIT* exon 18, *TP53* exon 10, *MET* exon 19, *GNAQ* exon 5, and *PDGFRA* exon 18 as well as the five targets as a multiplex. Lane 1 shows a control with the UPS adapter and UPS primer but untreated by Cas9/sgRNA. It shows background of varied length resulting from the ligation of UPS adapters to available ends in the DNA population. Most of the sample DNA is large in size (average size ~100 kb) and only produces a primer extension product in off-target regions. However, there is also a small population of off-target DNA that is short enough to be amplified adding to the overall background present. Without the addition of the UPS adapter (Lane 2) little non-specific background is observed from the UPS primers alone. Lanes 4–8 show results from CAMP enrichment for each noted locus and Lane 9 shows results of all five targets in a multiplex. The gel result of CAMP on the five targets and multiplex (Lanes 4–9) are difficult to distinguish from the background shown in Lane 1.

Therefore, to investigate the enrichment for the loci targeted by CAMP, a qPCR analysis was performed for each site. The qPCR enrichment results are shown in Fig 1B and were calculated by comparing the signal from a qPCR probe set within the targeted locus with a qPCR probe set outside a region of interest. As expected, little enrichment is observed in the samples untreated with Cas9/sgRNA (Fig 1B, Samples 1–3). These results suggest that any background exhibited in the gel (Fig 1B, Lane 1) was due to a low level of non-specific extension and amplification products across the genome. Samples 4–8 list the significant enrichment of the five targeted loci, ranging from 1.5x10^5^ to 3.1x10^6^ fold, and Sample 9 lists the enrichment of the five target multiplex which ranges from 2.6x10^4^ to 7.9x10^5^ fold.

### Demonstration of multiplexed enrichment with cCAMP on long human genomic DNA

**Fig 2:**
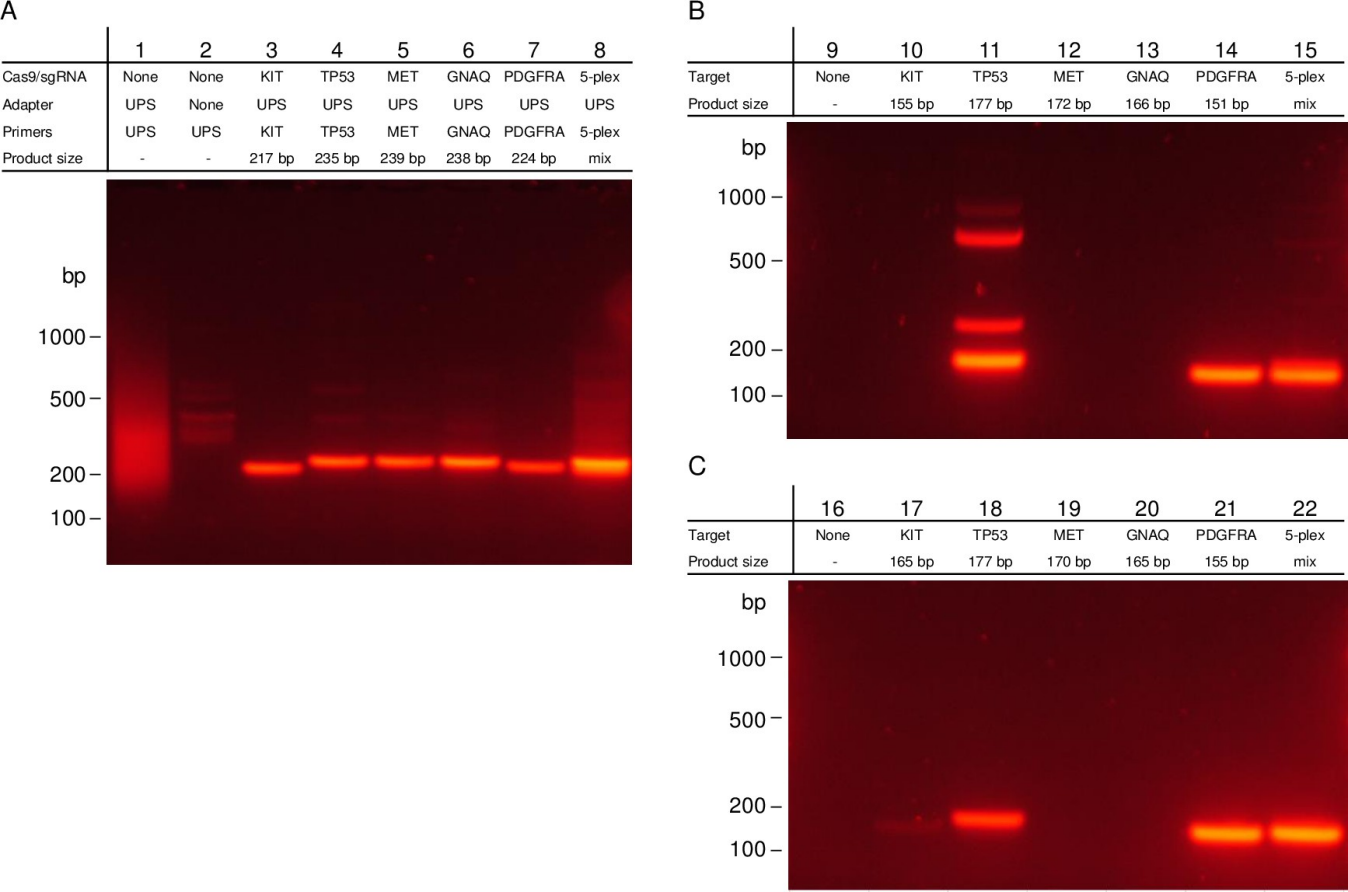
Comparison of gel electrophoresis results for cCAMP and sequence specific PCR on five individual targets and a multiplex. (A) Gel electrophoresis results of cCAMP. Lanes 1 and 2 show controls without Cas9/sgRNA with and without UPS adapters; amplification is complete with UPS primers. Lanes 3–7 show the results of cCAMP targeting *KIT* exon 18, *TP53* exon 10, *MET* exon 19, *GNAQ* exon 5, and *PDGFRA* exon 18, respectively. Lane 8 shows the five targets as a multiplex. Noted product lengths include 42 bp for the addition of the UPS adapters. (B) Gel results of sequence specific PCR designed to have the same melting temperature as the UPS component of the chimeric primers (63°C). (C) Gel results of sequence specific PCR designed to have the same melting temperature as the full chimeric primers (66°C). Sequence specific PCR were performed under same conditions as cCAMP (annealing temperature 65°C) and include a no template control in the first lane of each gel.

To decrease the low-level whole genome background demonstrated in CAMP, we next investigated the addition of several bases of target specificity to the 3’-end of the universal primers to produce chimeric primers; due to the UPS these chimeric primers maintain similar annealing temperatures across different targets. This method, termed cCAMP, uses a similar procedure to CAMP by first cutting with two Cas9/sgRNA complexes (S1 Table) and then ligating UPS adapters. However, it then uses chimeric primers (S2 Table) to amplify. cCAMP was first tested on the five targets described above as both individual reactions and as a multiplex. Fig 2A shows these results: Lanes 3–7 show individual results for targets in *KIT* exon 18, *TP53* exon 10, *MET* exon 19, *GNAQ* exon 5, and *PDGFRA* exon 18 and Lane 8 shows the results of the multiplex of the five targets. All show a single band at the noted size for the targeted sequence plus adapters, and the identity of each band was confirmed by an on-target qPCR probe set which showed enrichments of 4.5 x10^7^–2.1 x10^8^ fold for individual targets and 4.7x10^6^–4.8x10^7^ for the multiplex when compared to an off-target probe set. As demonstrated by the single band in Lanes 3–8 on the gel and the increase in fold enrichment by qPCR, the cCAMP methodology was successful in increasing the specificity of amplification and overall enrichment when compared to the results shown for CAMP.

One of the significant advantages of this technology when compared to sequence specific PCR, is a single set of PCR conditions for all targets. To further demonstrate this comparison, two sets of sequence specific PCR primers were designed for each target. In one, the primers were designed to have the same melting temperature as the UPS primer used (63°C, shown in Fig 2B), and in the other, the primers were designed to have the same melting temperature as the average of the full chimeric primers (66°C, shown in Fig 2C). In both cases, using the single condition (annealing temperature 65°C) used in cCAMP to amplify the individual targets and the multiplex only yield single band products in some cases. In Fig 2B, only *PDGFRA* exon 14 and the multiplex show single bands. In Fig 2C, *TP53* exon 10, *PDGFRA* exon 14 and the 5-plex show an intense single band and *KIT* exon 18 shows a faint band.

**Fig 3:**
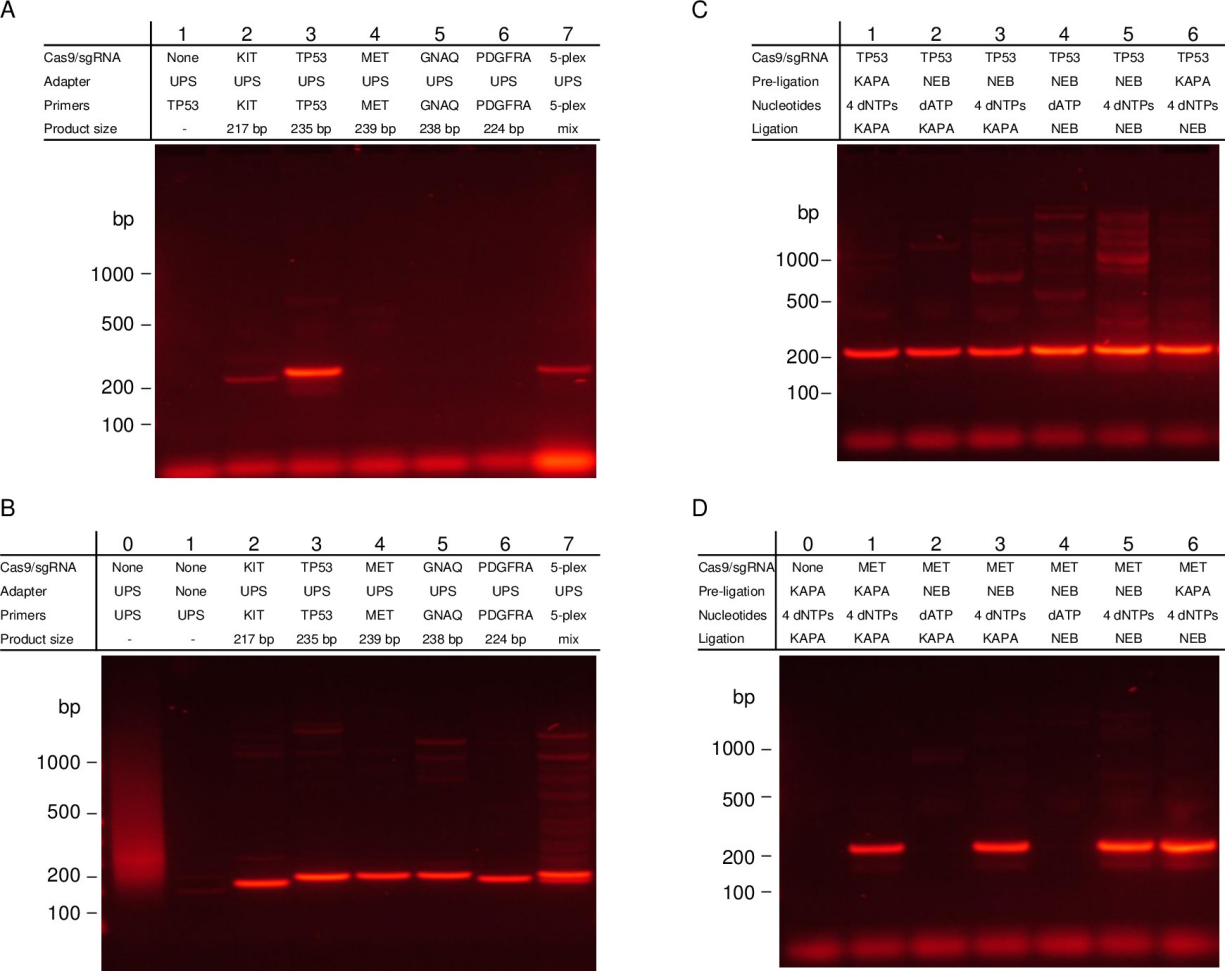
Evidence for Cas9/sgRNA produced staggered cut. (A) Gel electrophoresis results of cCAMP with dA-tailing only before ligation. Lane 1 shows a control reaction without Cas9 with UPS adapter and the *TP53* chimeric primers. Lanes 2–6 show results targeting *KIT* exon 18, *TP53* exon 10, *MET* exon 19, *GNAQ* exon 5, and *PDGFRA* exon 18, respectively. Lane 7 shows the five targets as a multiplex. (B) Gel electrophoresis results of cCAMP with dA-tailing module and added dCTP, dGTP and dTTP. Lanes 0 and 1 show control reactions without Cas9/sgRNA with and without UPS adapters with amplification complete with UPS primers. Lanes 2–6 show cCAMP results targeting *KIT* exon 18, *TP53* exon 10, *MET* exon 19, *GNAQ* exon 5, and *PDGFRA* exon 18, respectively. Lane 7 shows the five targets as a multiplex. (C) Analysis of components for the DNA pre-ligation preparation and ligation for *TP53* exon 10. (D) Analysis of components for the DNA pre-ligation preparation and ligation for *MET* exon 19. For (D) Lane 0 shows a control without Cas9 with UPS adapter and *MET* exon 19 primers with KAPA HyperPlus End Repair & A-Tailing Buffer (four dNTPs) and KAPA HyperPlus Ligation. For (B) and (D) Lane 1 shows cCAMP with KAPA HyperPlus End Repair & A-Tailing Buffer (four dNTPs) and KAPA HyperPlus Ligation, Lane 2 shows cCAMP with NEBNext Ultra II dA-tailing (dATP) and KAPA HyperPlus Ligation, Lane 3 shows cCAMP with NEBNext Ultra II dA-tailing with three additional dNTPs added (four dNTPs) and KAPA HyperPlus Ligation, Lane 4 shows cCAMP with NEBNext Ultra II dA-tailing (dATP) and NEBNext Ultra II Ligation, Lane 5 shows cCAMP with NEBNext Ultra II dA-tailing with three additional dNTPs added (four dNTPs) and NEB ligation, and Lane 6 shows cCAMP with KAPA HyperPlus End Repair & A-Tailing Buffer (four dNTPs) and NEBNext Ultra II Ligation. In all samples, Klenow (exo-) was included in pre-ligation reaction.

In the development of cCAMP, two protocols were tested to prepare the DNA for ligation and to ligate the UPS adapters. Initially, cCAMP was tested by preparing the DNA for ligation with Klenow (exo-) in KAPA HyperPlus End Repair & A-Tailing Buffer. The ligation was then completed with the KAPA HyperPlus Ligation Buffer and Ligase Enzyme. The buffer used had all four deoxynucleotide triphosphates (dNTPs) present, and the cCAMP results associated with this are shown in Fig 2A, discussed above. Fig 3A shows the results for the same targets, but with an alternative protocol for the pre-ligation and ligation steps. Here, the NEBNext dA-Tailing Module and the NEBNext Ultra II Ligation modules were used to dA-tail and ligate the adapters after treatment with Cas9/sgRNA. In NEBNext dA-Tailing Buffer, only dATP was present. Lanes 4–6 show complete loss of product for *MET* exon 19, *GNAQ* exon 5, and *PDGFRA* exon 18 and a weakening of the *KIT* exon 18 product with this alteration to the original protocol.

Recently, others have reported that the endonuclease activity of Cas9 produces staggered ends, contrary to the previous convention that Cas9/sgRNA produces blunt-end cuts [61–64]. We hypothesized that these staggered ends generated by Cas9/sgRNA are present during the cCAMP protocol and cause a loss of enrichment in several of the targets when the components for a fill-in repair are not provided before ligation. To investigate this hypothesis, the components for the pre-ligation and ligation steps were compared for *TP53* exon 10, which showed amplification with both protocols (Fig 3C), and for *MET* exon 19 which only showed amplification with the reagents purchased from Kapa Biosystems which had all four dNTPs (Fig 3D) present for repair. In each gel, Lane 1 shows the original method previously shown in Fig 2A with the KAPA HyperPlus End Repair & A-Tailing Buffer and KAPA HyperPlus Ligation and Lane 4 shows the method previously shown in Fig 3A with the NEB dA-tailing module and NEB ligation. Both were consistent with the previous results. Lanes 2 and 6 compare the ligation methods used by each method by combining the NEB dA-tailing step with the KAPA HyperPlus Ligation module (Lane 2) and the Kapa Biosystems end repair with the NEB ligation module (Lane 6). Lane 2 shows that enrichment is still lost in *MET* exon 19 with the dA-tailing module even when the KAPA HyperPlus Ligation is used, and Lane 6 has enrichment with the Kapa Biosystems end repair included even when the NEB ligation module is used. The results for *TP53* exon 10 still showed enrichment in both cases. Lanes 3 and 5 show results using the NEB dA-Tailing Module with the other three dNTPs (dCTP, dGTP, dTTP) added combined with the KAPA HyperPlus Ligation (Lane 3) and the NEBNext Ultra II Ligation (Lane 5). *TP53* exon 10 retains enrichment in both cases, and the enrichment in *MET* exon 19 is fully recovered when all four dNTPs are present pointing to a need to complete end repair in addition to dA-tailing prior to adapter ligation.

Fig 3B shows the cCAMP results of the five single targets and 5-plex completed with the NEBNext dA-Tailing Module with the additional three dNTPs added and the NEBNext Ultra II Ligation module used. Enrichment is observed in all lanes including *MET* exon 19, *GNAQ* exon 5, and *PDGFRA* exon 18 further confirming that for these targets a fill-in is needed due to the presence of staggered ends from the Cas9/sgRNA complex.

### Demonstration of multiplexed enrichment with cCAMP on a cfDNA model

**Fig 4:**
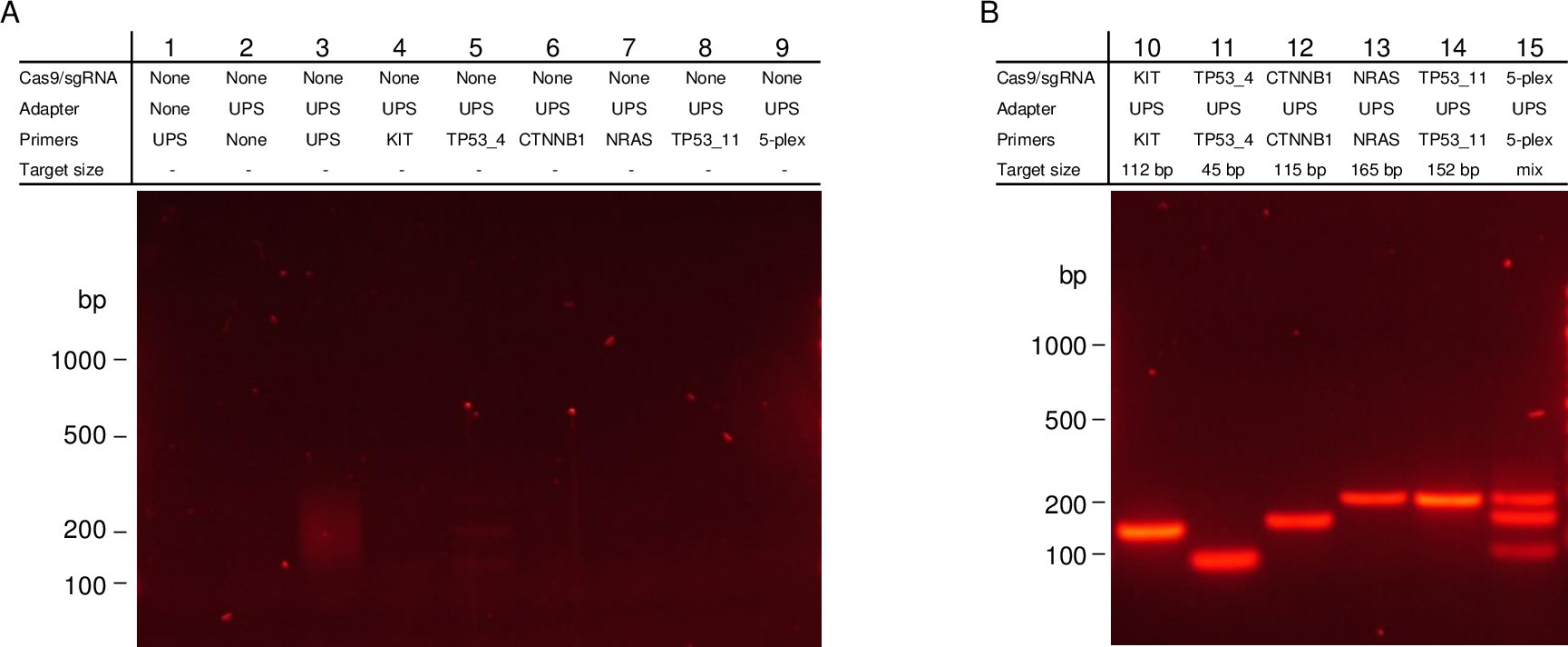
cCAMP enrichment from a cfDNA model. (A) Gel electrophoresis of cCAMP with a cfDNA model input control reaction results. Lane 1 shows a control without Cas9/sgRNA, without UPS adapters, and is amplified with UPS primers. Lane 2 shows a control without Cas9/sgRNA with UPS adapter but no primers. Lane 3 shows a positive control without Cas9/sgRNA, with UPS adapters, and is amplified with a UPS primer. Lanes 4–8 show the results of the cCAMP protocol without the addition of Cas9/sgRNA complexes but with UPS adapters and chimeric primers for *KIT* exon 18, *TP53* exon 4, *CTNNB1* exon 4, *NRAS* exon 4, and *TP53* exon 11, respectively. Lane 9 shows the results of the multiplex without Cas9/sgRNA. (B) Gel electrophoresis of cCAMP with a cfDNA model input. Lanes 10–14 show the cCAMP procedure, including treatment with Cas9/sgRNA complexes targeting *KIT* exon 18, *TP53* exon 4, *CTNNB1* exon 4, *NRAS* exon 4, and *TP53* exon 11, respectively. Lane 15 shows results of the five targets as a multiplex. Note, the sizes listed only include the length required between the two Cas9/sgRNA complexes and do not include the length of the UPS adapter.

We next demonstrated the application of cCAMP to a cfDNA model with an average fragment size of 166 bp for targeted enrichment. In order to extend the protocol from the long genomic DNA to the shorter length DNA in the cfDNA model, more specificity had to be introduced from the chimeric primer: for cCAMP with long input DNA a six base sequence specific region was used in the chimeric primer and for the cfDNA model a ten base sequence specific region was employed in the chimeric primer. Due to the length of the primer, new PCR conditions were developed for optimal enrichment, and the Cas9/sgRNA complexes were spaced less than 170 bp apart in order to effectively capture the smaller DNA fragments. Fig 4 shows the results of cCAMP on five targets and a 5-plex in a cfDNA model for *KIT* exon 18, *TP53* exon 4, *CTNNB1* exon 4, *NRAS* exon 4, and *TP53* exon 11. Fig 4A shows control reactions without Cas9/sgRNA: Lane 1 shows results with no adapter with UPS primer, Lane 2 shows results with UPS adapter and no primer, and Lane 3 shows results with UPS adapter and UPS primer. Lanes 4–9 are the results of cCAMP protocol performed for each of the targets listed with all components except the Cas9/sgRNA complex. No significant DNA signal is observed in any of these lanes except in Lane 3 which was expected to produce a smear due to non-specific binding of the UPS adapters and amplification with the non-chimeric UPS primer. Fig 4B shows cCAMP on the cfDNA model for *KIT* exon 18 (Lane 10), *TP53* exon 4 (Lane 11), *CTNNB1* exon 4 (Lane 12), *NRAS* exon 4 (Lane 13), and *TP53* exon 11 (Lane 14). It additionally shows cCAMP of all five targets as a multiplex in Lane 15. Each individual target lane shows one single PCR product with the expected size for the target plus 42 bp due to the UPS adapter, and the 5-plex shows three bands at approximately 200 bp due to *NRAS* exon 4 (207 bp) and *TP53* exon 11 (194 bp), approximately 160 bp due to *KIT* exon 18 (154 bp) and *CTNNB1* exon 4 (157 bp), and at approximately 90 bp due to *TP53* exon 4 (87 bp).

### Demonstration of mutation specific enrichment with TRACE (Post-PCR)

We next investigated the use of CRISPR for mutation specific enrichment. TRACE, the first mutation specific enrichment described in this report, uses Negative Enrichment [8] to afford single base discretion by using Cas9/sgRNA complexes to protect only the mutant allele from exonuclease digestion. As an initial investigation of this technology we tested TRACE on a PCR product designed with phosphorothioated primers such that only a single Cas9/sgRNA would be needed to provide protection of mutant alleles. Additionally, to simplify the proof of concept system further, mismatches were designed in the sgRNA to mimic mutations in the DNA.

**Fig 5:**
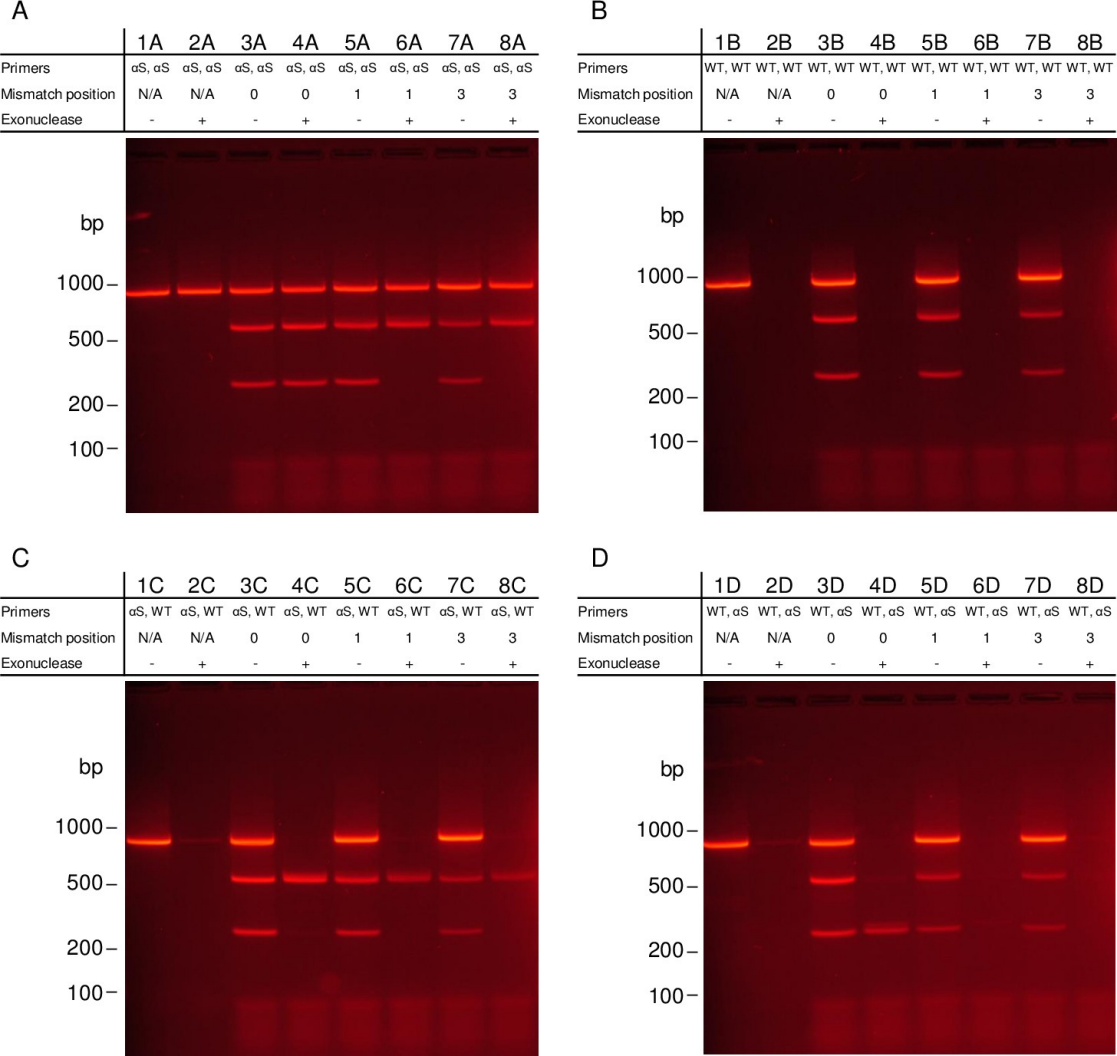
Gel electrophoresis results of mutation specific enrichment by TRACE on a series of phosphorothioated PCR products. (A) TRACE on an 820 bp PCR product around the *CFTR* F2 sgRNA site with both primers phosphorothioated. (B) TRACE on the 820 bp PCR product with both primers phosphorylated. (C) TRACE on the 820 bp PCR product with the forward primer phosphorothioated and the reverse primer phosphorylated. (D) TRACE of the 820 bp PCR product with the forward primer phosphorylated and the reverse primer phosphorothioated. In each gel, (A)—(D), Lanes 1 and 2 show controls without Cas9/sgRNA treatment without and with exonuclease. Lanes 3 and 4 show the results of Cas9/sgRNA treated reactions with a perfectly matched sgRNA without and with exonuclease treatment. Lanes 5 and 6 show the results of Cas9/sgRNA treated reactions with a mismatch in the sgRNA that produces a mismatch in the first position prior to the PAM site without and with exonuclease treatment. Lanes 7 and 8 show the results of Cas9/sgRNA treated reactions with a mismatch in the sgRNA that produces a mismatch in the third position prior to the PAM site without and with exonuclease treatment.

An 820 bp PCR product was designed around an sgRNA site (*CFTR* F2) within *CFTR* locus (S1 Table). The PCR product was amplified with phosphorothioated primers to protect one or both ends from lambda exonuclease (5’ to 3’ exonuclease activity) in four combinations of 5’-phosphorylated (wt) and 5’-phosphorothioated (αS) primers: forward: αS, reverse: αS (Fig 5A); forward: wt, reverse: wt (Fig 5B); forward αS, reverse: wt (Fig 5C); forward: wt, reverse: αS (Fig 5D). The 820 bp fragment was also designed such that upon cleavage by the Cas9/sgRNA complex it would separate into a 545 bp fragment (5’) and 275 bp fragment (3’) and the Cas9/sgRNA oriented so that the protospacer adjacent motif (PAM, 5’-NGG-3’) was facing the 3’-end. Fig 5 shows the gel electrophoresis results of the PCR products treated with Cas9/sgRNA with varying mismatches within the sgRNA sequence. Each PCR product was treated with several Cas9/sgRNA with and without exonuclease: one with a perfect match to the template DNA (Lanes 3 and 4), one with a single base mismatch that creates a mismatch to the DNA immediately before the PAM (Lanes 4 and 5), and one with a single base mismatch that creates a mismatch with the DNA three bases before the PAM (Lanes 7 and 8). Additionally, for each PCR product tested, a control without Cas9/sgRNA was added to show the effect of exonuclease on the products in the absence of Cas9/sgRNA protection (Lanes 1 and 2).

Interestingly, cutting is observed in all samples treated with Cas9/sgRNA, even those with mismatches (Lanes 3, 5, and 7 in Fig 5A–5D); the expected 545 bp and 275 bp fragment are observed, as well as some of the uncleaved starting material, due to the large number of genomic equivalents of PCR product added. Single base discretion is only observed through the protection from exonuclease. With a perfect match the Cas9/sgRNA protects both sides of the cut site, however asymmetric protection is indicated in samples with a mismatch. Here, the Cas9/sgRNA protects the 545 bp fragment which is on the PAM-distal side of the sgRNA, but not the 275 bp fragment which is on the same side of the cut site as the PAM site. This is most clearly supported in the samples with both sides phosphorothioated (Fig 5A), as these had additional protection of both ends from the phosphorothioated bases. With a perfect match, both fragments are protected and observed on the gel. However, with a mismatch, the 275 bp fragment is available for digestion (Lanes 6 and 8). For samples where both primers are phosphorylated (Fig 5B, even Lanes), no protection is indicated as expected. In samples with the 5’-end phosphorothioated (fragment towards PAM-distal side of the sgRNA), all lanes show protection of the 545 bp fragment only (Fig 5C, Lanes 4, 6, and 8). When the phosphorothioated bases are on the reverse primer, placing the protected end on the same side of the cut site as the PAM, only the perfect match shows protection (Fig 5D, Lane 4). The 275 bp fragment is protected by the perfect match of the Cas9/sgRNA complex and the 5’ phosphorothioated bases, but in the mismatched samples, there is no protection provided by the Cas9/sgRNA (Fig 5D, Lanes 6 and 8). Because this system results in protection of the 275 bp fragment only when there was a perfect match and the degradation of all other material, it was used in the subsequent analysis of additional targets.

**Fig 6:**
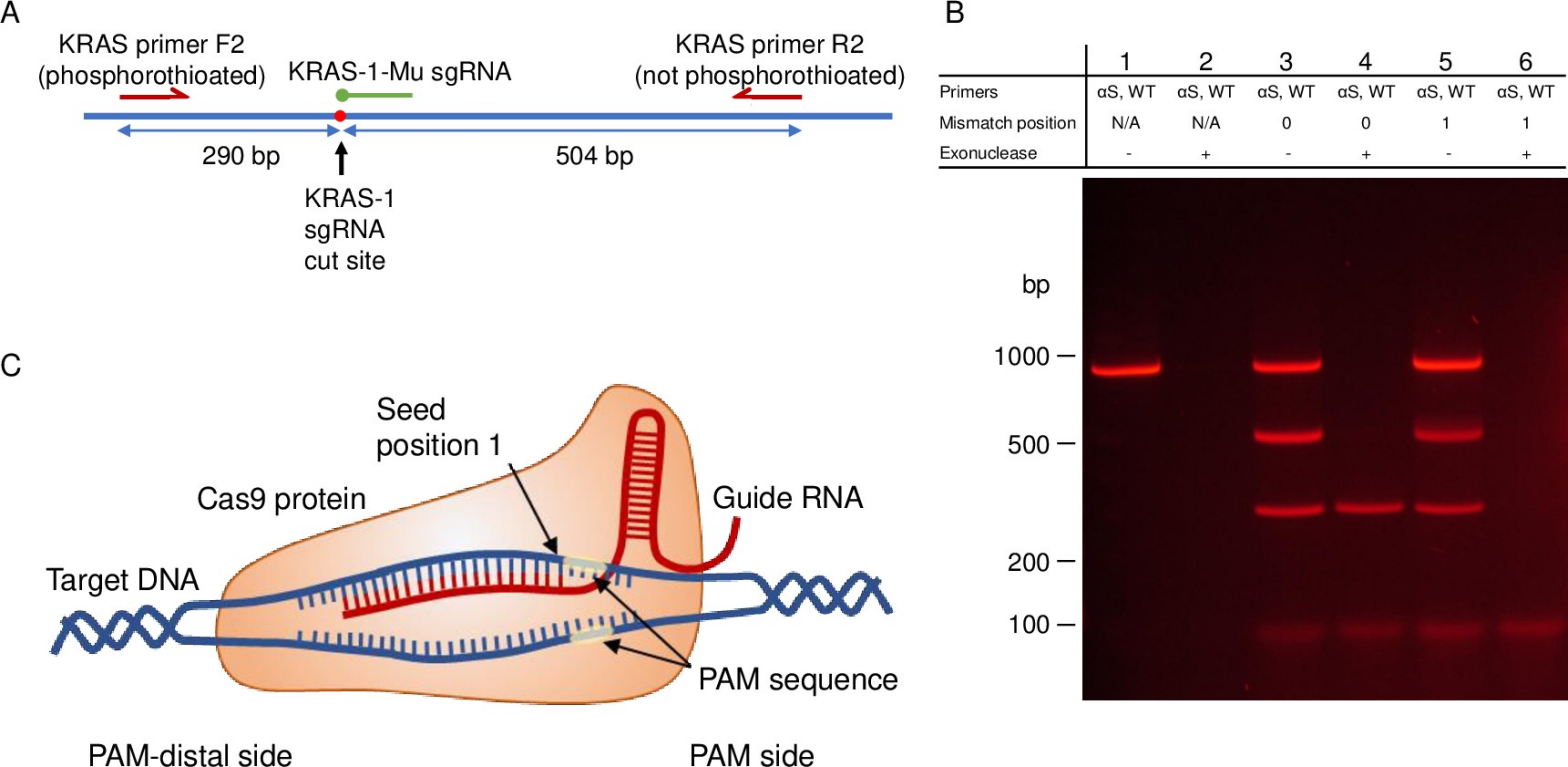
Gel electrophoresis results of mutation specific enrichment by TRACE on a phosphorothioated PCR product around the *KRAS* G12 locus. (A) Diagram of the PCR product designed around the *KRAS* G12 locus. (B) Results of TRACE performed on the 794 bp PCR product around *KRAS* G12 with the forward primer phosphorothioated. PCR product produced from normal human genomic DNA. Lanes 1 and 2 show controls without Cas9/sgRNA treatment without and with exonuclease treatment. Lanes 3 and 4 show results of Cas9/sgRNA treated reactions with an sgRNA that is a perfect match to the normal variant PCR product without and with exonuclease treatment. Lanes 5 and 6 show results of Cas9/sgRNA treated reactions with an sgRNA that matches the *KRAS* G12D mutation, producing a mismatch in the first position prior to the PAM site without and with exonuclease treatment. (C) Diagram of the Cas9/sgRNA complex.

To demonstrate the applicability of this system and to confirm our initial observation, a more clinically relevant locus was examined. A 794 bp PCR product was similarly amplified from normal human genomic DNA to contain the *KRAS* G12 locus in exon 2. The *KRAS* PCR product was synthesized with a forward phosphorothioated primer, and the sgRNA was designed so that the PAM was on the same side of the cut site as the phosphorothioated end with the *KRAS* G12D mutation position immediately before the PAM (S1 Table, Fig 6A and 6C). The 794 bp *KRAS* product was designed to produce a 290 bp fragment (5’, αS) and 504 bp fragment (3’, wt) upon cleavage with Cas9/sgRNA. The results of TRACE on the normal variant *KRAS* PCR product with and without exonuclease are shown in Fig 6B: without Cas9/sgRNA (Lanes 1 and 2), with Cas9/sgRNA with a perfect match to normal human genomic DNA (Lanes 3 and 4), and with Cas9/sgRNA with a match to the G12D mutation (chr12:25,245,350, C to T, hg38), forming a mismatch to the normal PCR product (Lanes 5 and 6).

As demonstrated with the *CFTR* F2 system above, single base discretion is observed in the protection by the Cas9/sgRNA from exonuclease, but not with cutting alone. When there is a perfect match (Lane 4), the 290 bp fragment is protected by the Cas9/sgRNA complex and the phosphorothioated primer, and the 504 bp fragment is digested from the 5’-end which lacks phosphorothioated bases. When the normal variant PCR product was treated with an sgRNA that would match the G12D mutation (Lane 6), no protection is observed.

### Demonstration of non-amplification based mutation specific enrichment with TRACE from long human genomic DNA

**Fig 7:**
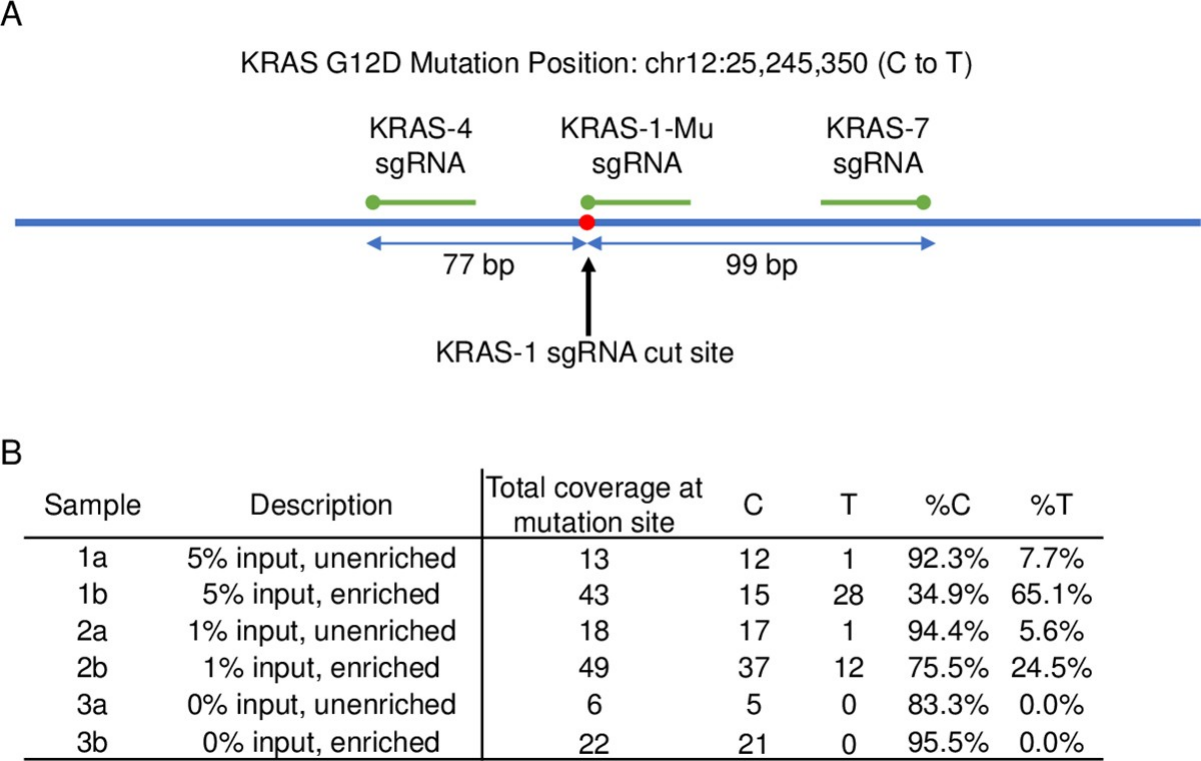
NGS results of mutation specific enrichment by TRACE targeting the *KRAS* G12D mutation with long human genomic input. (A) Schematic of the region around the *KRAS* G12D locus and placement of the sgRNA designed. (B) NGS results of long human genomic input with 5%, 1%, and 0% mutational frequency of the *KRAS* G12D mutation with (b) and without (a) TRACE enrichment.

Since the proof of concept studies described above showed single base discretion using TRACE, we next extended this method to long genomic DNA containing the *KRAS* G12D mutation. Additionally, the pre-PCR step was removed by using additional Cas9/sgRNA complexes to provide the required points of protection in place of the phosphorothioated primers. The initial demonstration of TRACE with no amplification was performed on DNA containing 5%,1%, and 0% of the *KRAS* G12D mutation. DNA was treated with three Cas9/sgRNA complexes and subsequently treated with exonuclease. In this study, the central sgRNA was designed to match the *KRAS* G12D mutation and designed such that the mutation was in the position directly before the PAM. The outer Cas9/sgRNA complexes were spaced 77 bp and 99 bp from the central Cas/sgRNA complex (Fig 7A). NGS analysis was completed on unenriched and enriched samples for each mutation frequency (Fig 7B). The 5% input mutation frequency was enriched from 7.7% to 65.1% of the *KRAS* G12D mutation and the 1% mutation frequency was enriched from 5.6% to 24.5% of the *KRAS* G12D mutation. These results are similar to estimated predictions based on a 95% exonuclease activity.

### Demonstration of non-amplification based mutation specific enrichment with TRACE from a cfDNA model

**Fig 8:**
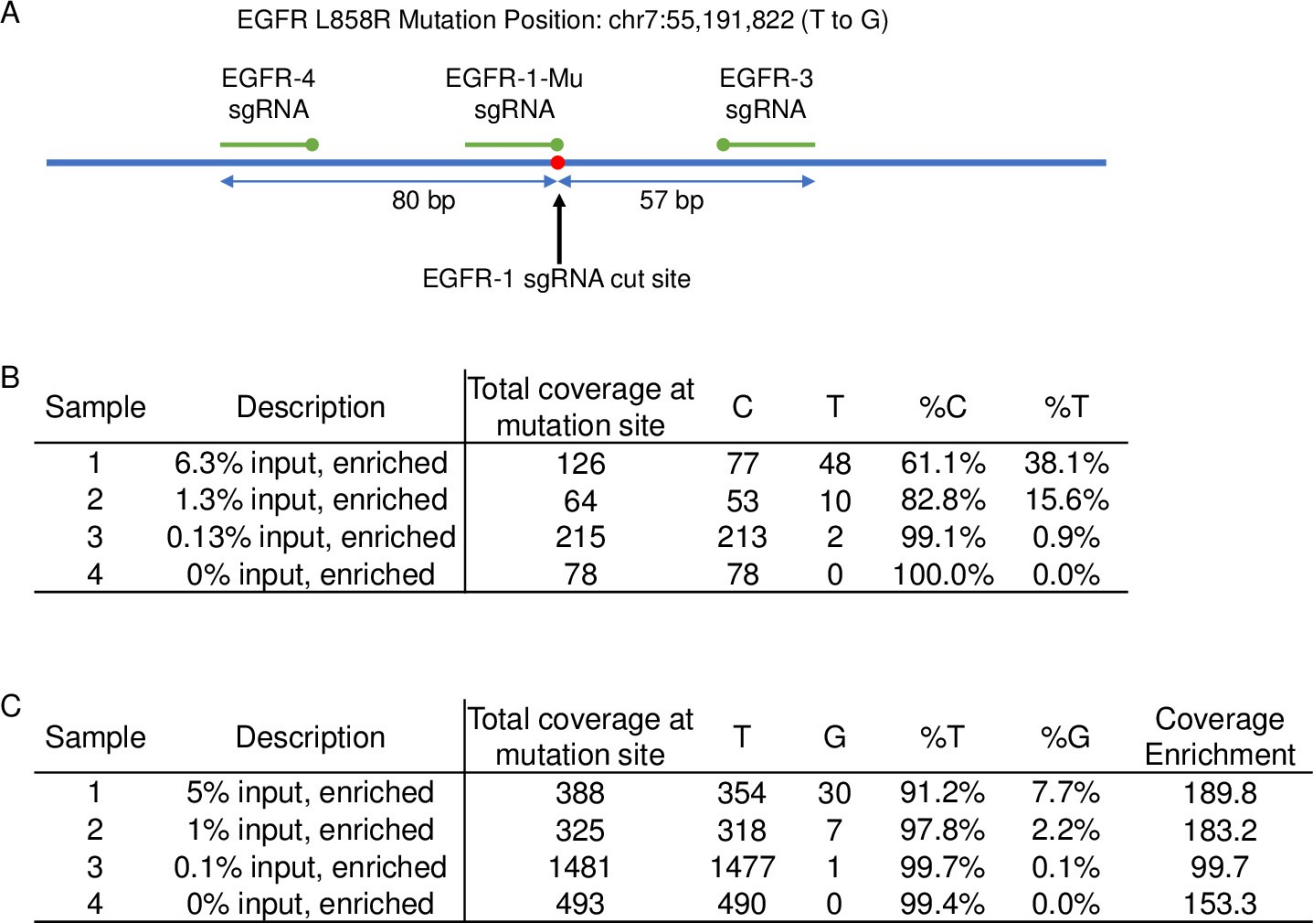
NGS results of multiplexed mutation specific enrichment by TRACE targeting the *KRAS* G12D and *EGFR* L858R mutations from a cfDNA model. **(**A) Schematic of the region around the *EGFR* L858R locus and placement of the sgRNA designed. (B) NGS results of TRACE enrichment of the *KRAS* G12D mutation from cfDNA input with 6.3%, 1.3%, 0.13%, and 0% mutation frequency input. (C) NGS results of TRACE enrichment of the *EGFR* L858R mutation from cfDNA input with 5%, 1%, 0.1%, and 0% mutation frequency input. The EGFR L858R mutation results in significant enrichment of the locus around the mutation site, and coverage enrichment was calculated based on the average coverage within the targeted locus divided by the average coverage outside the targeted locus.

We next demonstrated the application of TRACE to a cfDNA model and showed its ability to multiplex. To do this, the *KRAS* Cas9/sgRNA complexes used above were combined with three additional Cas9/sgRNA complexes that were designed to enrich for the *EGFR* L858R mutation (chr7:55,191,822 T to G, hg38). TRACE was then performed on a purchased model cfDNA with a series of known clinically relevant mutations including both *KRAS* G12D and *EGFR* L858R. A schematic of the *EGFR* L858R sgRNA is shown in Fig 8A. The central Cas9/sgRNA was designed such that only the *EGFR* L858R mutation had a PAM site and the outer Cas9/sgRNA were spaced 80 bp and 57 bp from the cut site.

The NGS results from the multiplexed enrichment using TRACE on the *KRAS* G12D locus are presented in Fig 8B and the *EGFR* L858R locus results are presented in Fig 8C. Enrichment of the *KRAS* G12D mutation was observed: the 6.3% input sample was enriched to 38.1%, the 1.3% input sample was enriched to 15.6%, and the 0.13% was enriched to 0.9%. It was expected that these enrichments were lower than the long DNA input samples, due to the fragmentation within the cfDNA.

The *EGFR* L858R data shows a small mutation specific enrichment: the 5% sample was enriched to 7.7% and the 1% sample was enriched to 2.2%. We hypothesize that this decrease in mutation specific enrichment is due to several other PAM sequences adjacent to the targeted sequence that the Cas9/sgRNA could have recognized in the normal variant. The target around the *EGFR* L858R locus, however, is considerably enriched. The 80 bp region between the 5’-Cas9/sgRNA complex and the central Cas9/sgRNA complex, was enriched to an average of 156-fold for the varied inputs. Coverage enrichment was calculated by the average coverage within the targeted locus divided by the average coverage outside the targeted locus.

### Demonstration of enrichment with cTRACE on human genomic DNA

**Fig 9:**
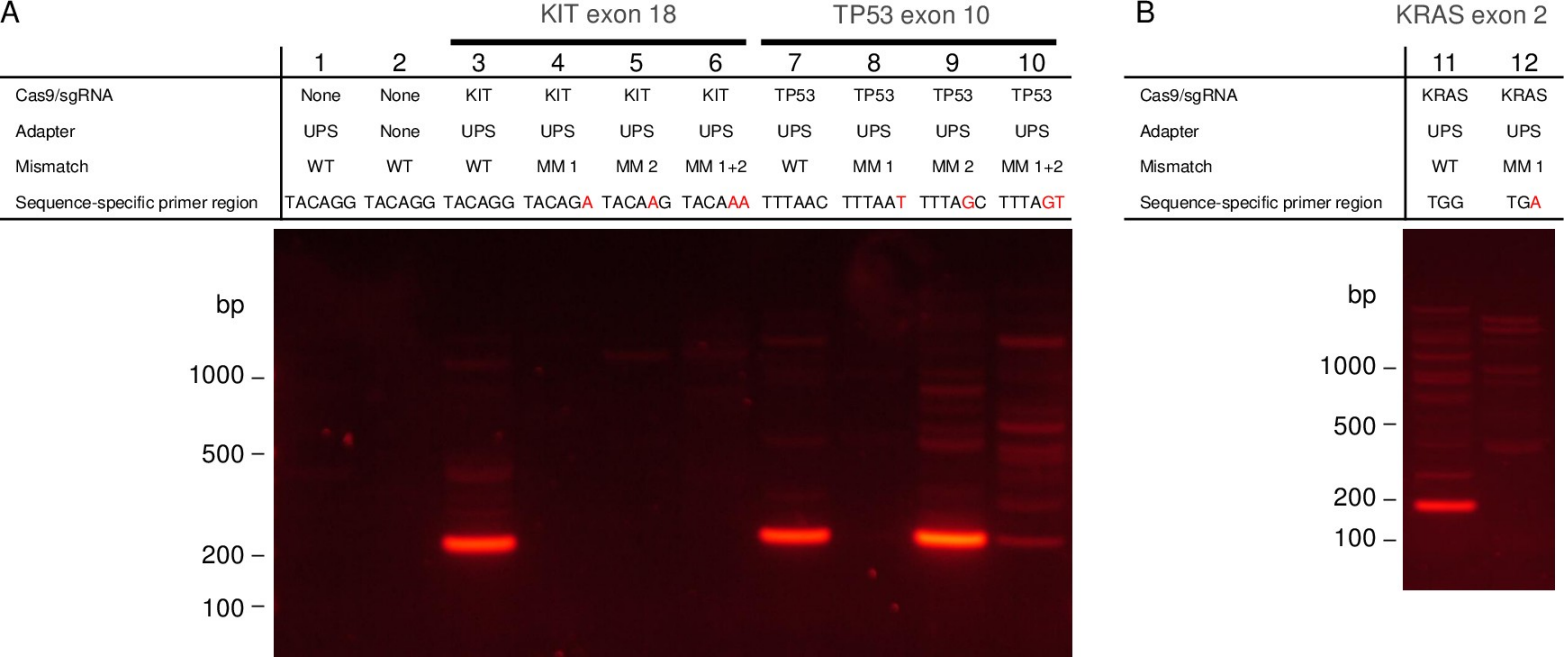
Gel electrophoresis showing single base discretion using cTRACE. (A) Demonstration of cTRACE on targets within *KIT* exon 18 and *TP53* exon 10 in normal human genomic DNA. Lanes 1 and 2 show controls without Cas9/sgRNA treatment with and without adapter, respectively, and amplified with *KIT* exon 18 chimeric primers. Lanes 3 and 7 show the results of cTRACE amplification with two perfectly matched primers for the two targets. Lanes 4 and 8 show the results of cTRACE amplification with one perfectly matched primer and one primer with a single mismatch at the 3’-end. Lanes 5 and 9 show the results of cTRACE amplification with one perfectly matched primer and one primer with a single mismatch in the second base from the 3’-end. Lanes 6 and 10 show the results of cTRACE amplification with one perfectly matched primer and one primer with two mismatches in the first and second position from the 3’-end. (B) Demonstration of cTRACE on *KRAS* exon 2 in normal human genomic DNA. Lane 1 shows enrichment found with a perfect matched primer and Lane 2 shows enrichment found with a single mismatch on the 3’-end of the primer that matches the *KRAS* G12D mutation.

TRACE is a powerful enrichment technology for single base mutations for applications that require the DNA in its native state. However, it requires the mutation to be in close proximity to a PAM sequence. To minimize this restriction, we developed cTRACE which is an amplification-based mutation specific enrichment much like cCAMP, discussed above. Like cCAMP, cTRACE uses Cas9/sgRNA complexes to ligate UPS adapters to a locus of interest and uses chimeric primers complementary to the UPS adapter and with several bases complementary to the targeted sequence added to the 3’-end. However, in addition to the target specificity, the 3’-end of the primer is also specific to a desired mutation. To model this technology, a series of chimeric primers were designed with varying mismatches to normal genomic DNA. Fig 9A shows results of cTRACE within *KIT* exon 18 and *TP53* exon 10. Each target was first cut with two Cas9/sgRNA (S1 Table) spaced 175 bp and 193 bp apart, respectively. Following ligation of universal adapters, the targets were amplified with one chimeric primer with a perfect match and one chimeric primer that was varied at the 3’-end to mimic mutations in the DNA (S2 Table). cTRACE results for *KIT* exon 18 and *TP53* exon 10 are shown with the varied primer as a perfect match (Lanes 3 and 7), as a single mismatch on 3’-end (Lanes 4 and 8), as a mismatch in the second base from the 3’-end (Lanes 5 and 9), and as two mismatches in both the first and second base from the 3’-end (Lanes 6 and 10). For the target in *KIT* exon 18 enrichment is only indicated for the primer with a perfect match, and for the target in *TP53* exon 10 there is enrichment for the perfect match, and discretion when a mismatch is present in the first base.

An additional demonstration of cTRACE was performed on a target within *KRAS* exon 2 around the site of the *KRAS* G12 locus (Fig 9B). To further draw a comparison between cTRACE and TRACE, the same Cas9/sgRNA was used to cut the DNA near the *KRAS* G12D mutation loci with an additional Cas9/sgRNA designed to cut 123 bp away. As shown in Fig 9B, enrichment is observed only with a perfect match between the primer and the normal variant DNA. In cTRACE, this enrichment is driven by the choice in primer rather than the specificity of the Cas9/sgRNA, as in TRACE. As demonstrated in the varied sequence specific component length of the chimeric primer between the targets in *KIT* exon 18 and *TP53* exon 10 and *KRAS* exon 2, cTRACE does not have significant limitations based on distance from the mutation site to an available PAM.

## Conclusions

Overall, the vast majority of investigations into the potential applications of CRISPR have focused on gene editing [1–3] and diagnostics [4–7]. Here, we have demonstrated an additional CRISPR-based technology: targeted and mutation specific DNA enrichment. This family of DNA enrichment techniques utilize the programmable sequence specificity of Cas9/sgRNA complexes to flank specific target sequences. Two amplification-based targeted enrichment methods have been shown. These methods use the Cas9/sgRNA to produce ligation sites in proximity to the region of interest to ligate universal adapters and amplify targets of interest. We first developed CAMP; in CAMP the amplification is completed using a UPS primer complementary to the UPS adapter alone. UPS adapters are ligated to many available ends throughout the genome. However, when the DNA input is long the proximity of the Cas9/sgRNA to one another affords a size selection that results in significant enrichment (~10^4^–10^6^).

As demonstrated, CAMP produces significant enrichment. However, there is a low level of non-specific background present across the genome due to primer extension from the ligated UPS adapters and some amplification products present from shorter DNA in the input sample. To further improve the enrichment by increasing the specificity of the amplification and to develop a technology that could also be applied to a cfDNA input, we next developed cCAMP. In cCAMP, Cas9/sgRNA are used to produce ligation sites and UPS adapters are ligated to available ends much like CAMP. However, the amplification is completed using a primer that contains both a region complementary to the UPS adapter as well as several bases of sequence specific to the targeted loci providing improved specificity. We have demonstrated this technology in both full length human genomic DNA and in a cfDNA model as five single targets and multiplexes. Analysis by gel shows a single band of the expected size for all targets. As we were developing these methods, we additionally found evidence that Cas9/sgRNA complexes do not always produce blunt-end cuts but also can produce a staggered cut site, like other recent reports have claimed [61–64].

Both of these targeted enrichment methodologies have a universal PCR condition for a multiplex of targets that requires little optimization for each additional target that is added. This overcomes many of the problems encountered when designing a large multiplex PCR for targeted enrichment [34]. While CAMP provides significant enrichment, it has low levels of non-specific background and is thus best suited for a platform like NGS, qPCR or digital PCR where the products can be distinguished over the background noise. cCAMP, however, can be easily visualized as a single target even on low resolution techniques like gel electrophoresis and can be coupled with any platform. cCAMP also can be used to enrich cfDNA, which typically presents significant analytical challenges and requires enrichment to produce clinically relevant assays for the early diagnosis of cancer and cancer recurrence monitoring [14–22]. Further work is currently ongoing by the authors of this manuscript to build on these methods by using a single Cas9/sgRNA complex for the enrichment of cfDNA, eliminating any limitation placed on the amplification by the short size of the input DNA. Additionally, the authors are pursuing the use of adapters that are resistant to exonuclease. Digestion of the background sequences will further eliminate any minor off-target amplification using CAMP and cCAMP methodologies, improving overall enrichment.

We also have demonstrated two mutation specific enrichment methodologies: TRACE and cTRACE. TRACE utilizes Negative Enrichment [8] by using the long residence time of Cas9/sgRNA [55,56] to provide steric inhibition from exonuclease which digests the DNA outside of the target loci as well as the normal untargeted allele. We have demonstrated this technology for both long DNA input as well as a cfDNA model. The 1% input mutational frequency of *KRAS* G12D was increased to 24.5% in long DNA and 15.6% in cfDNA. TRACE is a non-amplification based enrichment methodology; it thus has the advantage of leaving the native DNA intact for any desired studies of DNA mutations and associated epigenetic markers.

cTRACE uses similar methodology to cCAMP, however, it uses a chimeric primer with a mutation specific 3’-end to enrich the mutated allele preferentially over the normal variant. In the demonstrated proof of concept model cTRACE was shown to provide a high level of enrichment that is visible as a pure product on a gel for three targets. cTRACE, like cCAMP, is amplified with a universal set of conditions that require little optimization for every additional product thus enabling a large mutation specific multiplex of desired mutations. Additionally, cTRACE has more flexibility in the location of the mutation in reference to a PAM site than TRACE. The mutation location in cTRACE is dependent on the length of the sequence specific component of the chimeric primer, however, TRACE has a strict requirement that the mutation must be complementary to a base in the seed region directly next to the PAM site.

Along with our previous publication describing Negative Enrichment [8], this family of technologies represent an application of CRISPR to the enrichment of both long and short targeted loci as well as mutations. It additionally can be applied to both non-amplification and amplification-based applications. These methodologies do not require extensive optimization for each target and thus large genomic panels could be produced without extensive development.

## Supporting information

S1 Fig Scheme describing CAMP, cCAMP, cTRACE, and TRACE. (A) Scheme describing CAMP, cCAMP, and cTRACE. In all three processes, Cas9/sgRNA are used to cleave either side of a targeted locus (red). Universal UPS adapters (green) are then ligated and amplification is completed. CAMP uses primers that have complementarity to the UPS adapter only, cCAMP uses chimeric primers that have complementarity to the UPS adapter and several bases of target DNA, and cTRACE uses chimeric primers that have complementarity to the UPS adapter, several bases of target DNA, and specificity for a mutation (X). (B) Scheme describing TRACE. This method uses Cas9/sgRNA to protect targeted DNA (red) from exonuclease which digests off-target sequences (blue). Additionally, the protection provided by the Cas9/sgRNA gives single base discrimination to protect a single base mutation (X) while digesting the normal variant.
(TIF)

S1 Table Cas9/sgRNAs used in this study. (DOCX)

S2 Table DNA adapters and primers used in this study. (DOCX)

S3 Table qPCR probe sets used in this study. (DOCX)

## References

[1] Jinek M, Chylinski K, Fonfara I, Hauer M, Doudna JA, Charpentier E. A programmable dual-RNA-guided DNA endonuclease in adaptive bacterial immunity. Science. 2012;337(6096):816–21. 10.1126/science.1225829 22745249 PMC6286148

[2] Cong L, Ran FA, Cox D, Lin S, Barretto R, Habib N, et al Multiplex Genome Engineering Using CRISPR/Cas Systems. Science. 2013;339(6121):819–23. 10.1126/science.1231143 23287718 PMC3795411

[3] Mali P, Yang L, Esvelt KM, Aach J, Guell M, DiCarlo JE, et al RNA-Guided Human Genome Engineering via Cas9. Science. 2013;339(6121):823–6. 10.1126/science.1232033 23287722 PMC3712628

[4] Gootenberg JS, Abudayyeh OO, Lee JW, Essletzbichler P, Dy AJ, Joung J, et al Nucleic acid detection with CRISPR-Cas13a/C2c2. Science. 2017;356(6336):438–42. 10.1126/science.aam9321 28408723 PMC5526198

[5] Chen JS, Ma E, Harrington LB, Da Costa M, Tian X, Palefsky JM, et al CRISPR-Cas12a target binding unleashes indiscriminate single-stranded DNase activity. Science. 2018;360(6387):436–9. 10.1126/science.aar6245 29449511 PMC6628903

[6] Myhrvold C, Freije CA, Gootenberg JS, Abudayyeh OO, Metsky HC, Durbin AF, et al Field-deployable viral diagnostics using CRISPR-Cas13. Science. 2018;360(6387):444–8. 10.1126/science.aas8836 29700266 PMC6197056

[7] Gootenberg JS, Abudayyeh OO, Kellner MJ, Joung J, Collins JJ, Zhang F. Multiplexed and portable nucleic acid detection platform with Cas13, Cas12a, and Csm6. Science. 2018;360(6387):439–44. 10.1126/science.aaq0179 29449508 PMC5961727

[8] Stevens RC, Steele JL, Glover WR, Sanchez-Garcia JF, Simpson SD, O'Rourke D, et al A novel CRISPR/Cas9 associated technology for sequence-specific nucleic acid enrichment. PLoS One. 2019;14(4):e0215441 10.1371/journal.pone.0215441 30998719 PMC6472885

[9] Ballester LY, Luthra R, Kanagal-Shamanna R, Singh RR. Advances in clinical next-generation sequencing: target enrichment and sequencing technologies. Expert Rev Mol Diagn. 2016;16(3):357–72. 10.1586/14737159.2016.1133298 26680590

[10] Hyman DM, Taylor BS, Baselga J. Implementing Genome-Driven Oncology. Cell. 2017;168(4):584–99. 10.1016/j.cell.2016.12.015 28187282 PMC5463457

[11] Huggett JF, Cowen S, Foy CA. Considerations for Digital PCR as an Accurate Molecular Diagnostic Tool. Clin Chem. 2015;61(1):79–88. 10.1373/clinchem.2014.221366 25338683

[12] Serratì S, De Summa S, Pilato B, Petriella D, Lacalamita R, Tommasi S, et al Next-generation sequencing: advances and applications in cancer diagnosis. OncoTargets Ther. 2016;9:7355–65. 10.2147/OTT.S99807 27980425 PMC5144906

[13] Green DM. Improving health care and laboratory medicine: the past, present, and future of molecular diagnostics. Proc (Bayl Univ Med Cent). 2005;18(2):125–9. 10.1080/08998280.2005.11928050 16200160 PMC1200712

[14] Gabriel E, Bagaria SP. Assessing the Impact of Circulating Tumor DNA (ctDNA) in Patients With Colorectal Cancer: Separating Fact From Fiction. Front Oncol. 2018;8:297 10.3389/fonc.2018.00297 30128304 PMC6088154

[15] Tie J, Wang Y, Tomasetti C, Li L, Springer S, Kinde I, et al Circulating tumor DNA analysis detects minimal residual disease and predicts recurrence in patients with stage II colon cancer. Sci Transl Med. 2016;8(346):346ra92–ra92. 10.1126/scitranslmed.aaf6219 27384348 PMC5346159

[16] Volik S, Alcaide M, Morin RD, Collins C. Cell-free DNA (cfDNA): Clinical Significance and Utility in Cancer Shaped By Emerging Technologies. Mol Cancer Res. 2016;14(10):898–908. 10.1158/1541-7786.MCR-16-0044 27422709

[17] Yang N, Li Y, Liu Z, Qin H, Du D, Cao X, et al The characteristics of ctDNA reveal the high complexity in matching the corresponding tumor tissues. BMC Cancer. 2018;18:319 10.1186/s12885-018-4199-7 29566644 PMC5865353

[18] Bardelli A, Pantel K. Liquid Biopsies, What We Do Not Know (Yet). Cancer Cell. 2017;31(2):172–9. 10.1016/j.ccell.2017.01.002 28196593

[19] Siravegna G, Marsoni S, Siena S, Bardelli A. Integrating liquid biopsies into the management of cancer. Nat Rev Clin Oncol. 2017;14:531–48. 10.1038/nrclinonc.2017.14 28252003

[20] Bronkhorst AJ, Ungerer V, Holdenrieder S. The emerging role of cell-free DNA as a molecular marker for cancer management. Biomol Detect and Quantif. 2019;17:100087 10.1016/j.bdq.2019.100087 30923679 PMC6425120

[21] Diaz LA, Bardelli A. Liquid Biopsies: Genotyping Circulating Tumor DNA. J Clin Oncol. 2014;32(6):579–86. 10.1200/JCO.2012.45.2011 24449238 PMC4820760

[22] Mader S, Pantel K. Liquid Biopsy: Current Status and Future Perspectives. Oncol Res and Treat. 2017;40(7–8):404–8. 10.1159/000478018 28693023

[23] García-García G, Baux D, Faugère V, Moclyn M, Koenig M, Claustres M, et al Assessment of the latest NGS enrichment capture methods in clinical context. Sci Rep. 2016;6:20948 10.1038/srep20948 26864517 PMC4750071

[24] Samorodnitsky E, Jewell BM, Hagopian R, Miya J, Wing MR, Lyon E, et al Evaluation of Hybridization Capture Versus Amplicon-Based Methods for Whole-Exome Sequencing. Hum Mutat. 2015;36(9):903–14. 10.1002/humu.22825 26110913 PMC4832303

[25] Gray PN, Dunlop CLM, Elliott AM. Not All Next Generation Sequencing Diagnostics are Created Equal: Understanding the Nuances of Solid Tumor Assay Design for Somatic Mutation Detection. Cancers (Basel). 2015;7(3):1313–32. 10.3390/cancers7030837 26193321 PMC4586770

[26] Zakrzewski F, Gieldon L, Rump A, Seifert M, Grützmann K, Krüger A, et al Targeted capture-based NGS is superior to multiplex PCR-based NGS for hereditary BRCA1 and BRCA2 gene analysis in FFPE tumor samples. BMC Cancer. 2019;19:396 10.1186/s12885-019-5584-6 31029168 PMC6487025

[27] Mertes F, Elsharawy A, Sauer S, Van Helvoort JMLM, Van Der Zaag PJ, Franke A, et al Targeted enrichment of genomic DNA regions for next-generation sequencing. Brief Funct Genomics. 2011;10(6):374–86. 10.1093/bfgp/elr033 22121152 PMC3245553

[28] Kozarewa I, Armisen J, Gardner AF, Slatko BE, Hendrickson CL. Overview of Target Enrichment Strategies. Curr Protoc Mol Biol. 2015;112(1):7.21.1–23. 10.1002/0471142727.mb0721s112 26423591

[29] Mamanova L, Coffey AJ, Scott CE, Kozarewa I, Turner EH, Kumar A, et al Target-enrichment strategies for next-generation sequencing. Nat Methods. 2010;7(2):111–8. 10.1038/nmeth.1419 20111037

[30] Bodi K, Perera AG, Adams PS, Bintzler D, Dewar K, Grove DS, et al Comparison of Commercially Available Target Enrichment Methods for Next-Generation Sequencing. Biomol Tech. 2013;24(2):73–86. 10.7171/jbt.13-2402-002 23814499 PMC3605921

[31] Lu W, Zhu M, Chen Y, Bai Y. A novel approach to improving hybrid capture sequencing targeting efficiency. Mol Cell Probes. 2019;46:101424 10.1016/j.mcp.2019.101424 31336168

[32] Finotti A, Allegretti M, Gasparello J, Giacomini P, Spandidos DA, Spoto G, et al Liquid biopsy and PCR-free ultrasensitive detection systems in oncology (Review). Int J Oncol. 2018;53(4):1395–434. 10.3892/ijo.2018.4516 30085333 PMC6086621

[33] Dobosy JR, Rose SD, Beltz KR, Rupp SM, Powers KM, Behlke MA, et al RNase H-dependent PCR (rhPCR): improved specificity and single nucleotide polymorphism detection using blocked cleavable primers. BMC biotechnol. 2011;11:80 10.1186/1472-6750-11-80 21831278 PMC3224242

[34] Elnifro EM, Ashshi AM, Cooper RJ, Klapper PE. Multiplex PCR: optimization and application in diagnostic virology. Clin Microbiol Rev. 2000;13(4):559–70. 10.1128/cmr.13.4.559-570.2000 11023957 PMC88949

[35] Wu LR, Chen SX, Wu Y, Patel AA, Zhang DY. Multiplexed enrichment of rare DNA variants via sequence-selective and temperature-robust amplification. Nat Biomed Eng. 2017;1(9):714–23. 29805844 10.1038/s41551-017-0126-5 PMC5969535

[36] Loree JM, Kopetz S, Raghav KPS. Current companion diagnostics in advanced colorectal cancer; getting a bigger and better piece of the pie. J Gastrointest Oncol. 2017;8(1):199–212. 10.21037/jgo.2017.01.01 28280626 PMC5334060

[37] Bottema CDK, Sommer SS. PCR amplification of specific alleles: Rapid detection of known mutations and polymorphisms. Mutat Res.1993;288(1):93–102. 10.1016/0027-5107(93)90211-w 7686270

[38] Little S. Amplification-Refractory Mutation System (ARMS) Analysis of Point Mutations. Curr Protoc Hum Genet. 1995;7(1):9.8.1–12. 10.1002/0471142905.hg0908s07 18428319

[39] Beltz K, Tsang D, Wang J, Rose S, Bao Y, Wang Y, et al A High-Performing and Cost-Effective SNP Genotyping Method Using rhPCR and Universal Reporters. Adv Biosci Biotechnol. 2018;09:497–512.

[40] Broude NE, Zhang L, Woodward K, Englert D, Cantor CR. Multiplex allele-specific target amplification based on PCR suppression. Proc Natl Acad Sci U S A. 2001;98(1):206–11. 10.1073/pnas.98.1.206 11136256 PMC14569

[41] Offin M, Chabon JJ, Razavi P, Isbell JM, Rudin CM, Diehn M, et al Capturing Genomic Evolution of Lung Cancers through Liquid Biopsy for Circulating Tumor DNA. J Oncol. 2017;2017:4517834 10.1155/2017/4517834 28392802 PMC5368362

[42] Shendure J, Ji H. Next-generation DNA sequencing. Nat Biotechnol. 2008;26:1135–45. 10.1038/nbt1486 18846087

[43] Stetson D, Ahmed A, Xu X, Nuttall BRB, Lubinski TJ, Johnson JH, et al Orthogonal Comparison of Four Plasma NGS Tests With Tumor Suggests Technical Factors are a Major Source of Assay Discordance. JCO Precis Oncol. 2019;3:1–9. 10.1200/PO.18.00191 35100678

[44] Gu W, Crawford ED, O'Donovan BD, Wilson MR, Chow ED, Retallack H, et al Depletion of Abundant Sequences by Hybridization (DASH): using Cas9 to remove unwanted high-abundance species in sequencing libraries and molecular counting applications. Genome Biol. 2016;17:41 10.1186/s13059-016-0904-5 26944702 PMC4778327

[45] Hardigan AA, Roberts BS, Moore DE, Ramaker RC, Jones AL, Myers RM. CRISPR/Cas9-targeted removal of unwanted sequences from small-RNA sequencing libraries. Nucleic Acids Res. 2019;47(14):e84 10.1093/nar/gkz425 31165880 PMC6698666

[46] Quan J, Langelier C, Kuchta A, Batson J, Teyssier N, Lyden A, et al FLASH: a next-generation CRISPR diagnostic for multiplexed detection of antimicrobial resistance sequences. Nucleic Acids Res. 2019;47(14):e83 10.1093/nar/gkz418 31114866 PMC6698650

[47] Watson CM, Crinnion LA, Hewitt S, Bates J, Robinson R, Carr IM, et al Cas9-based enrichment and single-molecule sequencing for precise characterization of genomic duplications. Lab Invest. 2019;100:135–46. 10.1038/s41374-019-0283-0 31273287 PMC6923135

[48] Gilpatrick T, Lee I, Graham JE, Raimondeau E, Bowen R, Heron A, et al Targeted nanopore sequencing with Cas9-guided adapter ligation. Nat Biotechnol. 2020;38(4):433–8. 10.1038/s41587-020-0407-5 32042167 PMC7145730

[49] Stangl C, de Blank S, Renkens I, Westera L, Verbeek T, Valle-Inclan JE, et al Partner independent fusion gene detection by multiplexed CRISPR-Cas9 enrichment and long read nanopore sequencing. Nat Commun. 2020;11:2861 10.1038/s41467-020-16641-7 32504042 PMC7275081

[50] Hafford-Tear NJ, Tsai Y-C, Sadan AN, Sanchez-Pintado B, Zarouchlioti C, Maher GJ, et al CRISPR/Cas9-targeted enrichment and long-read sequencing of the Fuchs endothelial corneal dystrophy–associated TCF4 triplet repeat. Genet Med. 2019;21(9):2092–102. 10.1038/s41436-019-0453-x 30733599 PMC6752322

[51] Giesselmann P, Brändl B, Raimondeau E, Bowen R, Rohrandt C, Tandon R, et al Analysis of short tandem repeat expansions and their methylation state with nanopore sequencing. Nat Biotechnol. 2019;37(12):1478–81. 10.1038/s41587-019-0293-x 31740840

[52] Lee J, Lim H, Jang H, Hwang B, Lee JH, Cho J, et al CRISPR-Cap: multiplexed double-stranded DNA enrichment based on the CRISPR system. Nucleic Acids Res. 2018;47(1):e1. 10.1093/nar/gky820 PMC6326800 30215766

[53] Slesarev A, Viswanathan L, Tang Y, Borgschulte T, Achtien K, Razafsky D, et al CRISPR/Cas9 targeted CAPTURE of mammalian genomic regions for characterization by NGS. Sci Rep. 2019;9:3587 10.1038/s41598-019-39667-4 30837529 PMC6401131

[54] Shuber AP, Grondin VJ, Klinger KW. A simplified procedure for developing multiplex PCRs. Genome Res. 1995;5(5):488–493. 10.1101/gr.5.5.488 8808470

[55] Ma H, Tu LC, Naseri A, Huisman M, Zhang S, Grunwald D, et al CRISPR-Cas9 nuclear dynamics and target recognition in living cells. J Cell Biol. 2016;214(5):529–37. 10.1083/jcb.201604115 27551060 PMC5004447

[56] Sternberg SH, Redding S, Jinek M, Greene EC, Doudna JA. DNA interrogation by the CRISPR RNA-guided endonuclease Cas9. Nature. 2014;507(7490):62–7. 10.1038/nature13011 24476820 PMC4106473

[57] Li H. Aligning sequence reads, clone sequences and assembly contigs with BWA-MEM. arXiv:13033997v1 [q-bioGN]. 2013.

[58] Li H, Handsaker B, Wysoker A, Fennell T, Ruan J, Homer N, et al The Sequence Alignment/Map format and SAMtools. Bioinformatics. 2009;25(16):2078–9. 10.1093/bioinformatics/btp352 19505943 PMC2723002

[59] Quinlan AR, Hall IM. BEDTools: a flexible suite of utilities for comparing genomic features. Bioinformatics. 2010;26(6):841–2. 10.1093/bioinformatics/btq033 20110278 PMC2832824

[60] Kent WJ, Sugnet CW, Furey TS, Roskin KM, Pringle TH, Zahler AM, et al The human genome browser at UCSC. Genome Res. 2002;12(6):996–1006. 10.1101/gr.229102 12045153 PMC186604

[61] Shou J, Li J, Liu Y, Wu Q. Precise and Predictable CRISPR Chromosomal Rearrangements Reveal Principles of Cas9-Mediated Nucleotide Insertion. Mol Cell. 2018;71(4):498–509. 10.1016/j.molcel.2018.06.021 30033371

[62] Zuo Z, Liu J. Cas9-catalyzed DNA Cleavage Generates Staggered Ends: Evidence from Molecular Dynamics Simulations. Sci Reports. 2016;6:37584 10.1038/srep37584 27874072 PMC5118739

[63] Li Y, Park AI, Mou H, Colpan C, Bizhanova A, Akama-Garren E, et al A versatile reporter system for CRISPR-mediated chromosomal rearrangements. Genome Biol. 2015;16(1):111 10.1186/s13059-015-0680-7 26018130 PMC4465146

[64] Lemos BR, Kaplan AC, Bae JE, Ferrazzoli AE, Kuo J, Anand RP, et al CRISPR/Cas9 cleavages in budding yeast reveal templated insertions and strand-specific insertion/deletion profiles. Proc Natl Acad Sci U S A. 2018;115(9):E2040–47. 10.1073/pnas.1716855115 29440496 PMC5834694

