# Supplementary figures and images for: Novel CRISPR-based sequence specific enrichment methods for target loci and single base mutations
Source: PLoS One. 2020 Dec 23;15(12):e0243781. doi: 10.1371/journal.pone.0243781 (PMC7757808; doi:10.1371/journal.pone.0243781)

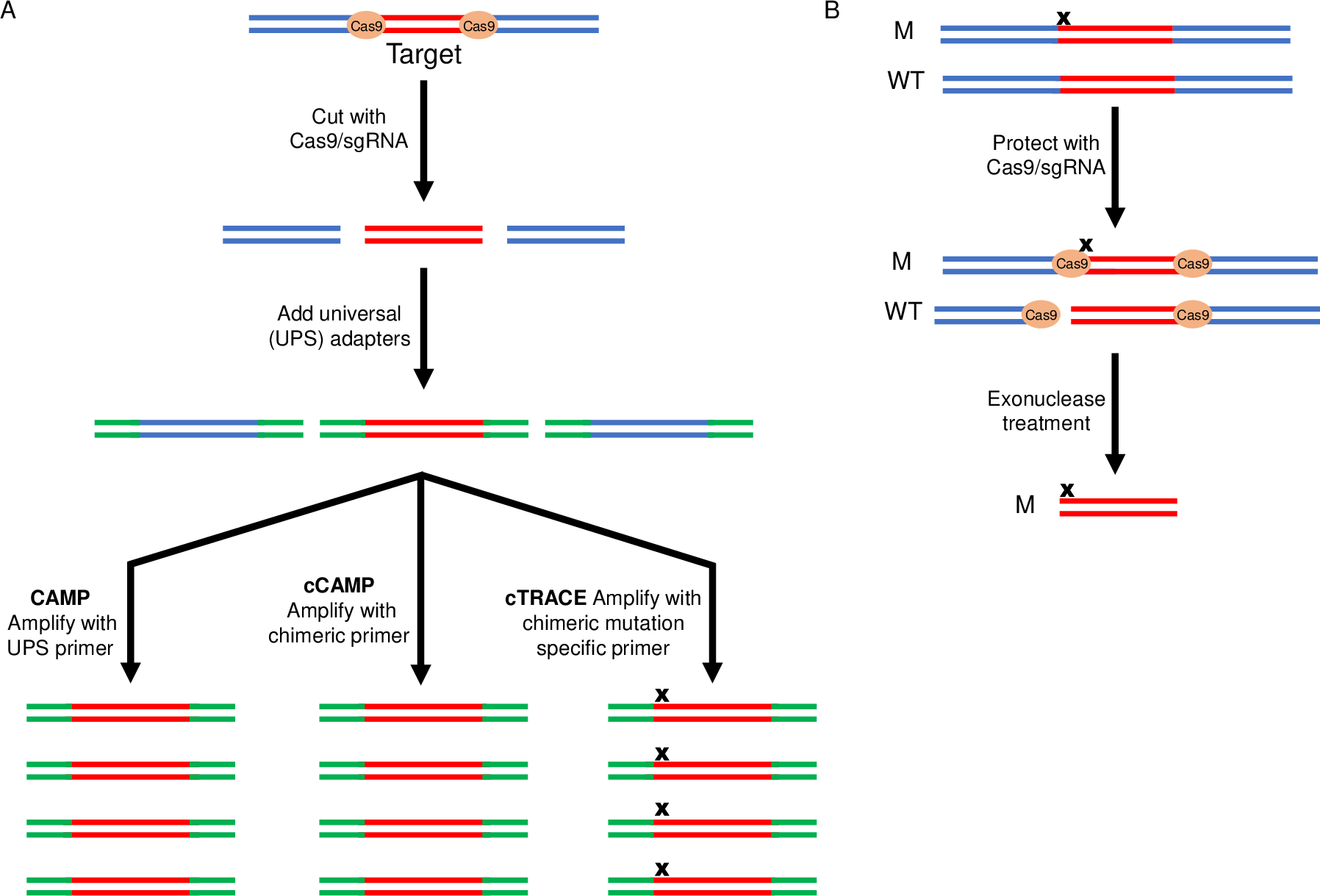

Supplement: S1 Fig — (A) Scheme describing CAMP, cCAMP, and cTRACE. In all three processes, Cas9/sgRNA are used to cleave either side of a targeted locus (red). Universal UPS adapters (green) are then ligated and amplification is completed. CAMP uses primers that have complementarity to the UPS adapter only, cCAMP uses chimeric primers that have complementarity to the UPS adapter and several bases of target DNA, and cTRACE uses chimeric primers that have complementarity to the UPS adapter, several bases of target DNA, and specificity for a mutation (X). (B) Scheme describing TRACE. This method uses Cas9/sgRNA to protect targeted DNA (red) from exonuclease which digests off-target sequences (blue). Additionally, the protection provided by the Cas9/sgRNA gives single base discrimination to protect a single base mutation (X) while digesting the normal variant. (TIF) [file pone.0243781.s001.tif]
