# Supplementary material for: Novel CRISPR-based sequence specific enrichment methods for target loci and single base mutations
Source: PLoS One. 2020 Dec 23;15(12):e0243781. doi: 10.1371/journal.pone.0243781 (PMC7757808; doi:10.1371/journal.pone.0243781)
Supplement: S1 Table — (DOCX) [file pone.0243781.s002.docx]

### S1 Table. Cas9/sgRNAs used in this study*

| **Experimental use** | **sgRNA or crRNA** | **Sequence (5' to 3')** |
| --- | --- | --- |
| Fig 1, 2, 4, 9 | sgRNA *KIT*_18_1 | CUCUGUUGUGCUUCUAUUACGUUUUAGAGCUAGAAAUAGCAAGUUAAAAUAAGGCUAGUCCGUUAUCAACUUGAAAAAGUGGCACCGAGUCGGUGCUUUU |
| Fig 1, 2, 9 | sgRNA *KIT*_18_2 | GCAUCUUCUUGAAGUUUCAUGUUUUAGAGCUAGAAAUAGCAAGUUAAAAUAAGGCUAGUCCGUUAUCAACUUGAAAAAGUGGCACCGAGUCGGUGCUUUU |
| Fig 1, 2, 9 | sgRNA *TP53*_10_1 | ACCUAGGAAGGCAGGGGAGUGUUUUAGAGCUAGAAAUAGCAAGUUAAAAUAAGGCUAGUCCGUUAUCAACUUGAAAAAGUGGCACCGAGUCGGUGCUUUU |
| Fig 1, 2, 9 | sgRNA *TP53*_10_2 | ACAGUACCUGAGUUAAAAGAGUUUUAGAGCUAGAAAUAGCAAGUUAAAAUAAGGCUAGUCCGUUAUCAACUUGAAAAAGUGGCACCGAGUCGGUGCUUUU |
| Fig 1, 2 | sgRNA *MET*_19_1 | AGGACAAAAAUUAUUACCCGGUUUUAGAGCUAGAAAUAGCAAGUUAAAAUAAGGCUAGUCCGUUAUCAACUUGAAAAAGUGGCACCGAGUCGGUGCUUUU |
| Fig 1, 2 | sgRNA *MET*_19_2 | AGUCAGAUGUGGUAAUGUAUGUUUUAGAGCUAGAAAUAGCAAGUUAAAAUAAGGCUAGUCCGUUAUCAACUUGAAAAAGUGGCACCGAGUCGGUGCUUUU |
| Fig 1, 2 | sgRNA *GNAQ*_5_1 | ACAAGUAAAUGAUAGAAAGUGUUUUAGAGCUAGAAAUAGCAAGUUAAAAUAAGGCUAGUCCGUUAUCAACUUGAAAAAGUGGCACCGAGUCGGUGCUUUU |
| Fig 1, 2 | sgRNA *GNAQ*_5_2 | CCUACAUCGACCAUUCUGCAGUUUUAGAGCUAGAAAUAGCAAGUUAAAAUAAGGCUAGUCCGUUAUCAACUUGAAAAAGUGGCACCGAGUCGGUGCUUUU |
| Fig 1, 2 | sgRNA *PDGFRA*_18_1 | AUCACGGUGGACACACUGCAGUUUUAGAGCUAGAAAUAGCAAGUUAAAAUAAGGCUAGUCCGUUAUCAACUUGAAAAAGUGGCACCGAGUCGGUGCUUUU |
| Fig 1, 2 | sgRNA *PDGFRA*_18_2 | UCACUUUAAUCUCUAAAGUCGUUUUAGAGCUAGAAAUAGCAAGUUAAAAUAAGGCUAGUCCGUUAUCAACUUGAAAAAGUGGCACCGAGUCGGUGCUUUU |
| Fig 4 | sgRNA *KIT*_L862 | UCUUUGGGACUGUUCUCUUUGUUUUAGAGCUAGAAAUAGCAAGUUAAAAUAAGGCUAGUCCGUUAUCAACUUGAAAAAGUGGCACCGAGUCGGUGCUUUU |
| Fig 4 | sgRNA *TP53*_P72_1 | ACCAGCAGCUCCUACACCGGGUUUUAGAGCUAGAAAUAGCAAGUUAAAAUAAGGCUAGUCCGUUAUCAACUUGAAAAAGUGGCACCGAGUCGGUGCUUUU |
| Fig 4 | sgRNA TP53_P72_2 | UGAAGCUCCCAGAAUGCCAGGUUUUAGAGCUAGAAAUAGCAAGUUAAAAUAAGGCUAGUCCGUUAUCAACUUGAAAAAGUGGCACCGAGUCGGUGCUUUU |
| Fig 4 | sgRNA *CNTTB1*_4_1 | CAGAGAAGGAGCUGUGGUAGGUUUUAGAGCUAGAAAUAGCAAGUUAAAAUAAGGCUAGUCCGUUAUCAACUUGAAAAAGUGGCACCGAGUCGGUGCUUUU |
| Fig 4 | sgRNA *CNTTB1*_4_2 | UUCACUCAAGAACAAGUAGCGUUUUAGAGCUAGAAAUAGCAAGUUAAAAUAAGGCUAGUCCGUUAUCAACUUGAAAAAGUGGCACCGAGUCGGUGCUUUU |
| Fig 4 | sgRNA *NRAS*_4_1 | CAGCCAAGACCAGACAGGUAGUUUUAGAGCUAGAAAUAGCAAGUUAAAAUAAGGCUAGUCCGUUAUCAACUUGAAAAAGUGGCACCGAGUCGGUGCUUUU |
| Fig 4 | sgRNA *NRAS*_4_2 | UUAAUCUGCUCCCUAAAAACGUUUUAGAGCUAGAAAUAGCAAGUUAAAAUAAGGCUAGUCCGUUAUCAACUUGAAAAAGUGGCACCGAGUCGGUGCUUUU |
| Fig 4 | sgRNA *TP53*_11_1 | GGGGGUGGGAGGCUGUCAGUGUUUUAGAGCUAGAAAUAGCAAGUUAAAAUAAGGCUAGUCCGUUAUCAACUUGAAAAAGUGGCACCGAGUCGGUGCUUUU |
| Fig 4 | sgRNA *TP53*_11_2 | AUGACAUCACAUGAGUGAGAGUUUUAGAGCUAGAAAUAGCAAGUUAAAAUAAGGCUAGUCCGUUAUCAACUUGAAAAAGUGGCACCGAGUCGGUGCUUUU |
| Fig 5 | sgRNA *CFTR* F2 | GGGGCUGGUAGUGUGAAGAUGUUUUAGAGCUAGAAAUAGCAAGUUAAAAUAAGGCUAGUCCGUUAUCAACUUGAAAAAGUGGCACCGAGUCGGUGCUUUU |
| Fig 5 | sgRNA *CFTR* F2 -M1 | GGGGCUGGUAGUGUGAAGAAGUUUUAGAGCUAGAAAUAGCAAGUUAAAAUAAGGCUAGUCCGUUAUCAACUUGAAAAAGUGGCACCGAGUCGGUGCUUUU |
| Fig 5 | sgRNA *CFTR* F2 -M3 | GGGGCUGGUAGUGUGAACAUGUUUUAGAGCUAGAAAUAGCAAGUUAAAAUAAGGCUAGUCCGUUAUCAACUUGAAAAAGUGGCACCGAGUCGGUGCUUUU |
| Fig 6, 7 | sgRNA *KRAS* -1-M | CUUGUGGUAGUUGGAGCUGAGUUUUAGAGCUAGAAAUAGCAAGUUAAAAUAAGGCUAGUCCGUUAUCAACUUGAAAAAGUGGCACCGAGUCGGUGCUUUU |
| Fig 7 | sgRNA *KRAS*-1 | CUUGUGGUAGUUGGAGCUGGGUUUUAGAGCUAGAAAUAGCAAGUUAAAAUAAGGCUAGUCCGUUAUCAACUUGAAAAAGUGGCACCGAGUCGGUGCUUUU |
| Fig 7 | sgRNA *KRAS*-4 | CGAAUAUGAUCCAACAAUAGGUUUUAGAGCUAGAAAUAGCAAGUUAAAAUAAGGCUAGUCCGUUAUCAACUUGAAAAAGUGGCACCGAGUCGGUGCUUUU |
| Fig 7 | sgRNA *KRAS*-7 | AUUAGAACAUGUCACACAUAGUUUUAGAGCUAGAAAUAGCAAGUUAAAAUAAGGCUAGUCCGUUAUCAACUUGAAAAAGUGGCACCGAGUCGGUGCUUUU |
| Fig 8 | sgRNA *EGFR*-4 | UCGCUUGGUGCACCGCGACCGUUUUAGAGCUAGAAAUAGCAAGUUAAAAUAAGGCUAGUCCGUUAUCAACUUGAAAAAGUGGCACCGAGUCGGUGCUUUU |
| Fig 8 | sgRNA *EGFR*-1 | UCAAGAUCACAGAUUUUGGGGUUUUAGAGCUAGAAAUAGCAAGUUAAAAUAAGGCUAGUCCGUUAUCAACUUGAAAAAGUGGCACCGAGUCGGUGCUUUU |
| Fig 8 | sgRNA *EGFR*-3 | UUACUUUGCCUCCUUCUGCAGUUUUAGAGCUAGAAAUAGCAAGUUAAAAUAAGGCUAGUCCGUUAUCAACUUGAAAAAGUGGCACCGAGUCGGUGCUUUU |
| Fig 9 | sgRNA *KRAS*-8 | CCUUUAUCUGUAUCAAAGAAGUUUUAGAGCUAGAAAUAGCAAGUUAAAAUAAGGCUAGUCCGUUAUCAACUUGAAAAAGUGGCACCGAGUCGGUGCUUUU |

*Purchased from Synthego (Redwood City, CA)
