# Supplementary material for: Novel CRISPR-based sequence specific enrichment methods for target loci and single base mutations
Source: PLoS One. 2020 Dec 23;15(12):e0243781. doi: 10.1371/journal.pone.0243781 (PMC7757808; doi:10.1371/journal.pone.0243781)
Supplement: S2 Table — (DOCX) [file pone.0243781.s003.docx]

**S2 Table. DNA adapters and primers used in this study***

| **Experimental use** | **Primer or adapter** | **Sequence (5' to 3')** |
| --- | --- | --- |
| Fig 1, 2, 3, 4, 9 | UPS adapter F | GCGGTCCCAAAAGGGTCAGT*T |
|  | UPS adapter R | [Phos]ACTGACCCTTTTGGGACCGC |
| Fig. 1, 2, 4 | UPS primer | GCGGTCCCAAAAGGGTCAGT |
| Fig. 2, 9 | *KIT* 18_F6 | GCGGTCCCAAAAGGGTCAGTTTACAGG |
| Fig. 2, 9 | *KIT*_18_R6 | GCGGTCCCAAAAGGGTCAGTTAAACTT |
| Fig. 2, 9 | *TP53*_10_F6 | GCGGTCCCAAAAGGGTCAGTTAGTAGG |
| Fig. 2, 9 | *TP53*_10_R6 | GCGGTCCCAAAAGGGTCAGTTTTTAAC |
| Fig. 2 | *KIT*_PCR_F1 | CAGGCTCGACTACCTGTGAAGTG |
| Fig. 2 | *KIT*_PCR_R1 | CTGAGTCTAATGAAGTTGTCTTTGGCA |
| Fig. 2 | *TP53*_PCR_F1 | CCAGGAAGGGGCTGAGGTCA |
| Fig. 2 | *TP53*_PCR_R1 | GGTACTGTGTATATACTTACTTCTCCCCC |
| Fig. 2 | *MET*_PCR_F1 | TGTCCTTTCTGTAGGCTGGATGA |
| Fig. 2 | *MET*_PCR_R1 | ACTTGGTGGTAAACTTTTGAGTTTGCA |
| Fig. 2 | *GNAQ*_PCR_F1 | TCAGATAATAAAATGATAATCCATTGCCT |
| Fig. 2 | *GNAQ*_PCR_R1 | GTAGGGGGCCAAAGGTCAGA |
| Fig. 2 | *PDGFRA*_PCR_F1 | CCACCGTGATCTGGCTGCTC |
| Fig. 2 | *PDGFRA*_PCR_R1 | TGAGCCTGACCAGTGAGGGA |
| Fig. 2 | *KIT*_PCR_F2 | CAGGCTCGACTACCTGTGAAGTGGA |
| Fig. 2 | *KIT*_PCR_R2 | AGAAGATGCTCTGAGTCTAATGAAGTTGT |
| Fig. 2 | *TP53*_PCR_F2 | CCAGGAAGGGGCTGAGGTCACTC |
| Fig. 2 | *TP53*_PCR_R2 | GGTACTGTGTATATACTTACTTCTCCCCCT |
| Fig. 2 | *MET*_PCR_F2 | TTGTCCTTTCTGTAGGCTGGATGAAA |
| Fig. 2 | *MET*_PCR_R2 | TGGTGGTAAACTTTTGAGTTTGCAGACT |
| Fig. 2 | *GNAQ*_PCR_F2 | CAGATAATAAAATGATAATCCATTGCCTGT |
| Fig. 2 | *GNAQ*_PCR_R2 | GTAGGGGGCCAAAGGTCAGAGAGA |
| Fig. 2 | *PDGFRA*_PCR_F2 | AGTGTGTCCACCGTGATCTGGCTG |
| Fig. 2 | *PDGFRA*_PCR_R2 | GCCTGACCAGTGAGGGAAGTGAGG |
| Fig. 2 | *MET*_19_F6 | GCGGTCCCAAAAGGGTCAGTTGTAATA |
| Fig. 2 | *MET*_19_R6 | GCGGTCCCAAAAGGGTCAGTTCATTAC |
| Fig. 2 | *GNAQ*_5_F6 | GCGGTCCCAAAAGGGTCAGTTTTCTAT |
| Fig. 2 | *GNAQ*_5_R6 | GCGGTCCCAAAAGGGTCAGTTAGAATG |
| Fig. 2 | *PDGFRA*_18_F6 | GCGGTCCCAAAAGGGTCAGTTAGTGTG |
| Fig. 2 | *PDGFRA*_18_R6 | GCGGTCCCAAAAGGGTCAGTTTTTAGA |
| Fig. 4 | *KIT*_18_F10 | GCGGTCCCAAAAGGGTCAGTTTACAGGCTCG |
| Fig. 4 | *KIT*_L862_R10 | GCGGTCCCAAAAGGGTCAGTTGAGAACAGCT |
| Fig. 4 | *TP53*_P72_F10 | GCGGTCCCAAAAGGGTCAGTTGTGTAGGAGC |
| Fig. 4 | *TP53*_P72_R10 | GCGGTCCCAAAAGGGTCAGTTCAGAGGCTGC |
| Fig. 4 | *CTNNB1_F10* | GCGGTCCCAAAAGGGTCAGTTCCACAGCTCC |
| Fig. 4 | *CTNNB1_R10* | GCGGTCCCAAAAGGGTCAGTTACTTGTTCTT |
| Fig. 4 | *NRAS*_4_F10 | GCGGTCCCAAAAGGGTCAGTTCTGTCTGGTC |
| Fig. 4 | *NRAS*_4_R10 | GCGGTCCCAAAAGGGTCAGTTTTTAGGGAGC |
| Fig. 4 | *TP53*_11_F10 | GCGGTCCCAAAAGGGTCAGTTAGTGGGGAAC |
| Fig. 4 | *TP53*_11_R10 | GCGGTCCCAAAAGGGTCAGTTCACTCATGTG |
| Fig. 5 | *CFTR* F2_F*_*wt | GTAATCGGCGGTGGAGGTAG |
| Fig. 5 | *CFTR* F2_F*_*αS | G*T*A*A*TCGGCGGTGGAGGTAG |
| Fig. 5 | CFTR F2_R_wt | TCCGTATTTCAGAAGACACCGG |
| Fig. 5 | *CFTR* F2_R*_*αS | T*C*C*G*TATTTCAGAAGACACCGG |
| Fig. 6 | *KRAS*_F2 | T*G*A*A*CATCATGGACCCTGACA |
| Fig. 6 | *KRAS*_R2 | CACTTAGAGGTGGGGGTCCA |
| Fig. 9 | *KIT*_18_F6_P1 | GCGGTCCCAAAAGGGTCAGTTTACAGA |
| Fig. 9 | *KIT*_18_F6_A1 | GCGGTCCCAAAAGGGTCAGTTTACAAG |
| Fig. 9 | *KIT*_18_F6_A1P1 | GCGGTCCCAAAAGGGTCAGTTTACAAA |
| Fig. 9 | *TP53*_10_R6_P1 | GCGGTCCCAAAAGGGTCAGTTTTTAAT |
| Fig. 9 | *TP53*_10_R6_A1 | GCGGTCCCAAAAGGGTCAGTTTTTAGC |
| Fig. 9 | *TP53*_10_R6_A1P1 | GCGGTCCCAAAAGGGTCAGTTTTTAGT |
| Fig. 9 | *KRAS*_2_1_F6 | GCGGTCCCAAAAGGGTCAGTTGAATGG |
| Fig. 9 | *KRAS*_G12_R6_wt | GCGGTCCCAAAAGGGTCAGTTTGG |
| Fig. 9 | *KRAS*_G12_R6_Mu | GCGGTCCCAAAAGGGTCAGTTTGA |

*Purchased from Integrated DNA Technologies (IDT, Skokie, Illinois)
