# Supplementary material for: Novel CRISPR-based sequence specific enrichment methods for target loci and single base mutations
Source: PLoS One. 2020 Dec 23;15(12):e0243781. doi: 10.1371/journal.pone.0243781 (PMC7757808; doi:10.1371/journal.pone.0243781)
Supplement: S3 Table — (DOCX) [file pone.0243781.s004.docx]

### S3 Table. qPCR probe sets used in this study*

| **Probe set** | **Sequences (5' to 3')** |
| --- | --- |
| CFTR 1 probe set 1 | Forward: TATGGGCTGTGCACTACTGG |
|  | Reverse: TGCTGAAGCAATACCACTCG |
|  | FAM Probe: GTCCACCTGGGCTAACTCCT |
| KIT_18 probe set 1 | Forward: GAAGTGGATGGCACCTGAAA |
|  | Reverse: CTAAAGAGAACAGCTCCCAAAGA |
|  | FAM Probe: ACGTTTGAAAGTGACGTCTGGTCCT |
| TP53_10 probe set 2 | Forward: TGAGGTCACTCACCTGGAG |
|  | Reverse: GAGATGTTCCGAGAGCTGAATG |
|  | FAM Probe: CTTGGAACTCAAGGATGCCCAGGC |
| MET_19 probe set 2 | Forward: CACAGTCAAGGTTGCTGATTT |
|  | Reverse: CTTTCCAAAGCCATCCACTTC |
|  | FAM Probe: AAACAGGTGCAAAGCTGCCAGT |
| GNAQ_5 probe set 1 | Forward: ATTGTCTGACTCCACGAGAAC |
|  | Reverse: GCCAAAGGTCAGAGAGAAGAA |
|  | FAM Probe: AGCGCTACTAGAAACATGATAGAGGTGACA |
| PDGFRA_18 probe set 3 | Forward: AAATTGTGAAGATCTGTGACTTTGG |
|  | Reverse: TGACCAGTGAGGGAAGTGA |
|  | FAM Probe: TGTGTCGAAAGGCAGTGTACGTCC |

*Purchased from Integrated DNA Technologies (IDT, Skokie, Illinois)
